# Distinct transcriptomic and epigenomic responses of mature oligodendrocytes during disease progression in a mouse model of multiple sclerosis

**DOI:** 10.1038/s41593-025-02100-3

**Published:** 2025-11-17

**Authors:** Chao Zheng, Bastien Hervé, Mandy Meijer, Leslie Ann Rubio Rodríguez-Kirby, André Ortlieb Guerreiro Cacais, Petra Kukanja, Mukund Kabbe, Tony Jimenez-Beristain, Tomas Olsson, Eneritz Agirre, Gonçalo Castelo-Branco

**Affiliations:** 1 Laboratory of Molecular Neurobiology, Department of Medical Biochemistry and Biophysics, Karolinska Institutet, Stockholm, Sweden; 2 Max Delbrück Center for Molecular Medicine in the Helmholtz Association (MDC Berlin), Berlin, Germany; 3 Neuroimmunology Unit, Department of Clinical Neuroscience, Center for Molecular Medicine, Karolinska Institute, Stockholm, Sweden

**Keywords:** Multiple sclerosis, Chromatin analysis, Animal disease models

## Abstract

Multiple sclerosis (MS) is a chronic autoimmune disease that targets mature oligodendrocytes (MOLs) and their myelin. MOLs are heterogeneous and can transition to immune-like states in MS. However, the dynamics of this process remain unclear. Here, we used single-cell multiome assay for transposase-accessible chromatin and RNA sequencing targeting oligodendroglia (OLG) from the experimental autoimmune encephalomyelitis (EAE) MS mouse model at multiple disease stages. We found that immune OLG states appear at early disease stages and persist to late stages, which can be consistent with epigenetic memory of previous neuroinflammation. Transcription factor activity suggested immunosuppression in OLG at early disease stages. Different MOLs exhibit differential responsiveness to EAE, with MOL2 exhibiting a stronger transcriptional immune response than MOL5/MOL6, and showed divergent responses at the epigenetic level during disease evolution. Our single-cell multiomic resource highlights dynamic and subtype-specific responses of OLG to EAE, which might be amenable to modulation in MS.

## Abstract (web summary)

Single-cell ATAC/RNA multiomic profiling was used to investigate how oligodendrocyte lineage cells respond across multiple disease stages within a mouse MS model, revealing early activation of immune genes along with oligodendrocyte subtype-specific responses.

## Main

Multiple sclerosis (MS) is an inflammatory autoimmune disease of the central nervous system (CNS)^1^. Oligodendrocytes (OLs) are the myelinating cells of the CNS, and their precursor cells (OL precursor cells (OPCs)) are present throughout the developing and adult CNS and are capable of differentiating into myelinating mature OLs (MOLs)^2^. MS has been generally viewed as primarily driven by T cells and B cells^3^. However, recent studies revealed the expression of immunomodulatory molecules in oligodendroglia (OLG) not only in MS but also in Alzheimer’s disease and aging^4–8^. These findings indicate the potential role of OLG in the modulation of immune responses within the CNS.

Single-cell/nucleus RNA sequencing (scRNA-seq) has been applied to reveal specific MOLs/OPC subpopulations in MS and experimental autoimmune encephalomyelitis (EAE)^4,9,10^. Assay for transposase-accessible chromatin using sequencing (ATAC-seq) is a high-throughput sequencing technique for assessing genome-wide chromatin accessibility^11^ and provides information on regions with open chromatin, which is required for gene expression^12^. By applying single-cell ATAC-seq (scATAC-seq) in combination with scRNA-seq independently, we previously found that a cohort of immune genes exhibit open chromatin in both control animals and in animals with EAE at peak disease, while their expression only increases in the context of EAE^13^. These studies were conducted with samples from single time points, at the peak of the disease in mouse EAE and from the late stage of disease in human postmortem MS tissue. Thus, the dynamics of OLG throughout the disease process have not been explored.

Here, we investigate the epigenomic and transcriptional dynamics of OPCs and MOLs over the course of EAE. We applied simultaneous single-cell multiome ATAC-seq and RNA-seq to OLG sorted by fluorescence-activated cell sorting (FACS) from male and female mice with EAE at three distinct time points: early, peak and late stages. At the early stage of EAE, a subset of genes involved in antigen presentation showed increased expression and chromatin accessibility in OPCs and MOLs, indicating that the induction of an immune-like state in OLG occurs before the formation of fully developed lesions. Moreover, chromatin accessibility at these genes remained highly open at the late stage of EAE, indicating either partial maintenance or an epigenetic memory of this immune-like state. Furthermore, specific MOL subtypes acquire different patterns of change in genes related to the immune response at both expression and chromatin accessibility levels over the course of disease. We observed that white matter-enriched^14^ MOL2 showed higher immune signatures than MOL5/MOL6, which in turn exhibited induction of chromatin accessibility in genes with regenerative pathways, particularly in the late stage. Our study provides a resource available for browsing at the University of California, Santa Cruz, Cell Browser and Genome Browser^15^ (https://olg-dyn-eae-multiome.cells.ucsc.edu) and a deeper understanding of OLG dynamics in the inflammatory demyelination mouse model of MS, offering insights into these cell populations as potential targets for immune modulation and myelin regeneration.

## Results

### Single-cell multiome analysis of OLG at different EAE stages

**Fig. 1:**
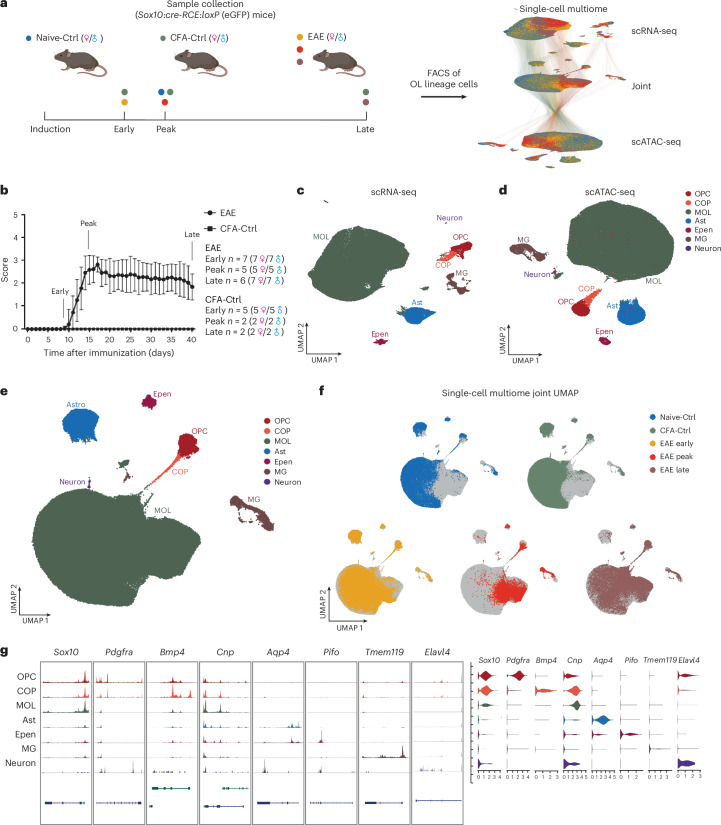
Single-cell multiome (RNA-seq + ATAC-seq) analysis of OLG in an EAE mouse model of MS. **a**, Schematic of the methodology used in animal model establishment and multiome sequencing (image created using BioRender); blue dots, Naive-Ctrl; green dots, CFA-Ctrl; yellow dots, EAE early stage; red dots, EAE peak stage; brown dots, EAE late stage. **b**, Clinical scores of the mice used in the study (EAE: 38 mice in 19 multiome experiments, CFA-Ctrl *n* = 9; data are shown as mean ± s.d.). For EAE from peak and late stages, only mice that had reached a score of 3 were used in this study. **c**,**d**, Uniform manifold approximation and projection (UMAP) of cells profiled with simultaneous scRNA-seq (**c**) and scATAC-seq (**d**). Cell types are identified according to marker genes; Ast, astrocytes; Epen, ependymal cells; MG, microglia; COP, committed OL precursor. **e**, Joint UMAP from the weighted nearest neighbors graph of scRNA-seq and scATAC-seq modalities colored by cell type. **f**, Joint UMAP with cells colored by conditions on top of a UMAP with all cells (in gray). **g**, Normalized chromatin accessibility (left) and log_2_ expression (right) of representative marker genes of each cell type.

**Extended Data Fig. 1:**
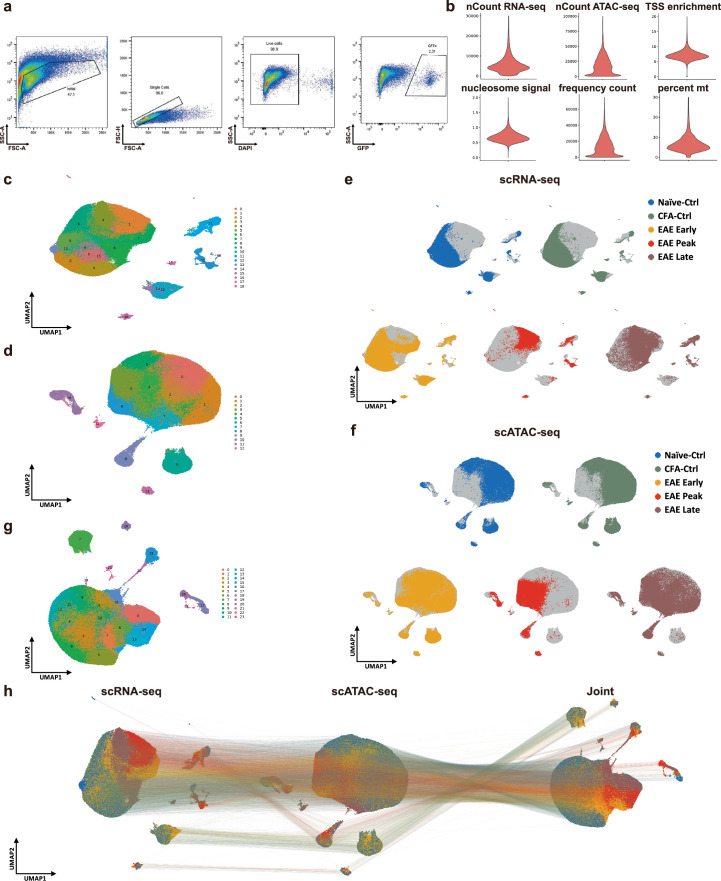
Single cell multiome of OLGs from the EAE model. **a**, Representative fluorescence-activated cell sorting (FACS) gating strategy with a sample from EAE peak stage. **b**, Quality control metrics after removing low quality cells of the multiome ATAC + gene expression sequencing data. **c**, **d**, UMAP with Louvain clustering algorithm based on gene expression (c) and chromatin accessibility (**d**) of 10x Genomics chromium multiome ATAC + gene expression. **e**,**f**, UMAP of the cells colored by condition on top of UMAP with all cells (in gray), based on gene expression data (**e**) and chromatin accessibility data (**f**). **g**, UMAP with Louvain clustering algorithm based on joint projection of gene expression and chromatin accessibility modalities. **h**, UMAP based on scRNA-seq data (left), scATAC-seq data (middle), and joint UMAP created by combining scRNA-seq and scATAC-seq nearest neighbors graphs (right), with lines connecting cells by single-cell barcodes across modalities.

We induced EAE in *Sox10:cre-RCE:loxP* (enhanced green fluorescent protein (eGFP)) transgenic mice^16,17^ with injection of emulsion containing the MOG_35–55_ immunogenic peptide in complete Freund’s adjuvant (CFA), followed by intraperitoneal injection of pertussis toxin. Spinal cord tissues from male and female mice induced with EAE were collected at three different stages: (1) early stage (days 8–9 after injection), (2) peak stage (days 14–15) and (3) late/chronic stage (days 37–40; Fig. 1a,b). Spinal cord samples from CFA-treated control (CFA-Ctrl) mice were also collected from the same stages, alongside spinal cord tissues from noninduced naive untreated control (Naive-Ctrl) mice. OLG were enriched based on eGFP by sorting by FACS (Extended Data Fig. 1a), after which we performed single-cell multiome RNA-seq and ATAC-seq (Fig. 1a). After sample-specific quality control filtering (Extended Data Fig. 1b and Methods), we obtained 156,205 cells. Louvain clustering was performed on the scRNA-seq (Extended Data Fig. 1c,e) and scATAC-seq datasets (Extended Data Fig. 1d,f). Neighbors’ graphs from both modalities were overlapped to generate a new joined projection (Fig. 1c–f and Extended Data Fig. 1g,h), annotated by cell type with marker gene expression and chromatin accessibility (Fig. 1g and Methods). As expected from the lineage tracing strategy, most of the cells were MOLs, OPCs and committed OL precursors. Nevertheless, other populations, in particular astrocytes and microglia, were also captured (Fig. 1c–e).

### Immune OLG states emerge early and persist in late EAE

**Fig. 2:**
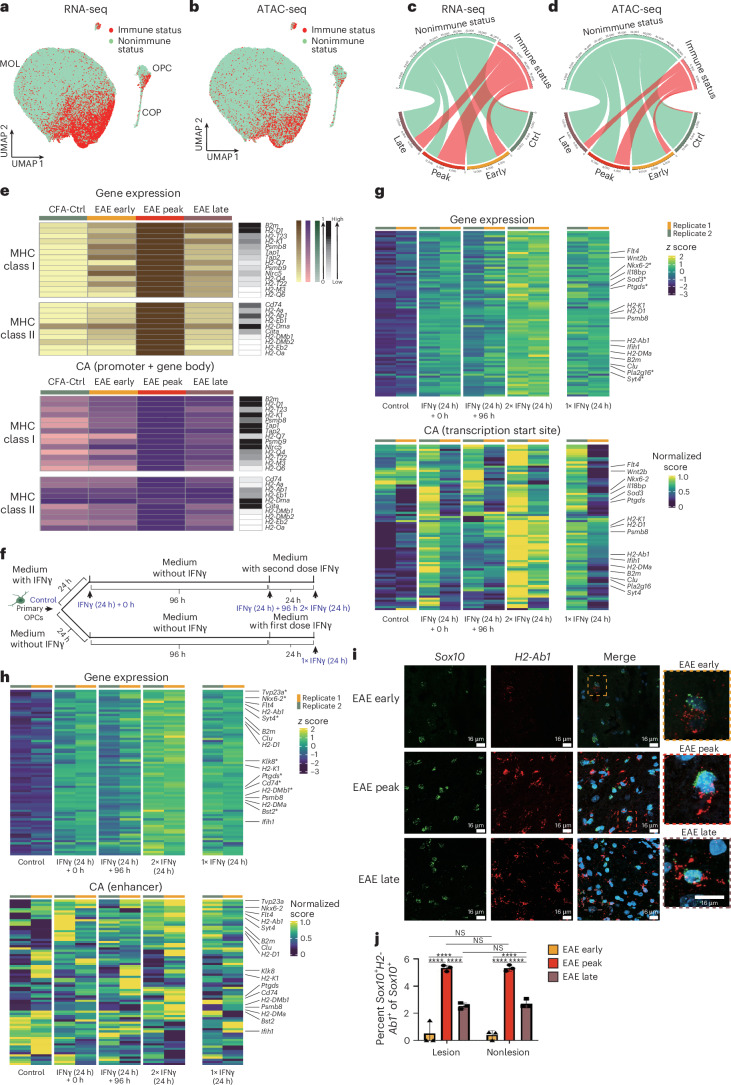
Transition to the imOLG states occurs at early stages of EAE and persists at late stages, consistent with epigenetic memory at a chromatin accessibility level. **a**,**b**, Joint UMAP with OLG from CFA-Ctrl mice and mice with EAE. Projection of cells with immune status (red) and nonimmune status (green) identified by gene expression (**a**) and chromatin accessibility (**b**). **c**,**d**, Circos plots showing the number of cells with (red) or without (green) immune status identified by gene expression (**c**) and chromatin accessibility (**d**; downsampled by time point). **e**, Heat maps of the expression (top) and chromatin accessibility (CA; bottom) of MHC class I (top) and MHC class II (bottom) genes at different stages. The black column on the right represents the gene raw counts. **f**, Schematic of the 1×/2× IFNγ treatment experiment on mouse primary OPCs. Arrows represent the time points of sample collection (image created using BioRender). **g**,**h**, Heat maps showing normalized gene expression (top) and chromatin accessibility signal in 1-kb windows around the TSS (bottom; **g**) at defined enhancer regions (Methods) in the same 1-kb windows (bottom; **h**) in mouse primary OPCs with the first and second doses of IFNγ. Genes plotted were upregulated following IFNγ treatment compared to control treatment (*P* < 0.05 and false discovery rate (FDR) < 0.05) and showed no changes in chromatin accessibility signal after the first treatment with IFNγ in the absence of this cytokine for 96 h (*P* > 0.05 and FDR > 0.05). Genes that were significantly upregulated following the second dose of IFNγ compared to the first dose of IFNγ are marked with an asterisk (*; *P* < 0.05 and FDR < 0.05), while the remaining genes presented *P* < 0.05 in this comparison. **i**, RNAscope ISH from early-, peak- and late-stage EAE mice marked with probes for *Sox10* (OLG) and *H2-Ab1* (MHC class II). **j**, Quantification of the percentages of *Sox10*^+^*H2-Ab1*^+^ cells out of *Sox10*^+^ cells in lesion and nonlesion areas. Statistical analyses were performed using a two-way ANOVA with a Tukey’s multiple comparisons test and adjusted *P* values; NS, *P* > 0.05; ****P < 0.0001; data are shown as mean ± s.d.; *n* = 3 independent experiments per condition. Source Data

We observed that a subset of MOLs transitioned to transcriptional/epigenomic states distinct from CFA-Ctrl at early stages of EAE, when lesions are just starting to develop (Fig. 1f and Extended Data Fig. 1e,f). Moreover, a minority of MOLs retained peak-stage-like profiles at late stages, while most MOLs transitioned to CFA-Ctrl-like states, suggesting a return to homeostasis (Fig. 1f and Extended Data Fig. 1e,f). Because disease-associated OLG states at peak EAE are characterized by chromatin accessibility and the expression of immune genes^4,13^, we investigated whether the observed transitions were driven by their immune status. We subsetted and reclustered OPCs and MOLs to identify those expressing immune-related genes^13^ (Fig. 2a and Methods). Immune OLG (imOLG) were hardly observed in CFA-Ctrl mice, but were found in mice with EAE, with a higher percentage present at the peak stage than in the early and late stages (early stage 26.80%, peak stage 66.91% and late stage 32.41%; Fig. 2c). Chromatin accessibility at the promoter/gene body of the same immune genes identified a lower number of imOLG than by immune gene expression (early 14.69%, peak stage 37.71% and late stage 17.81%; Fig. 2b,d). This is most likely due to some OLG from CFA-Ctrl and Naive-Ctrl animals exhibiting already primed chromatin accessibility in immune gene loci but with low or no expression^4,13^, which made the cutoff value of immune status depicted from gene chromatin accessibility higher than from expression. Thus, our data indicate that the transition of both OPCs and MOLs to immune-like states at epigenomic and transcriptional levels occurs at early stages of EAE, when lesions are just starting to develop, and persists at late stages, despite resolving inflammation.

### OLG MHC class I/MHC class II chromatin accessibility persists in late EAE

**Fig. 3:**
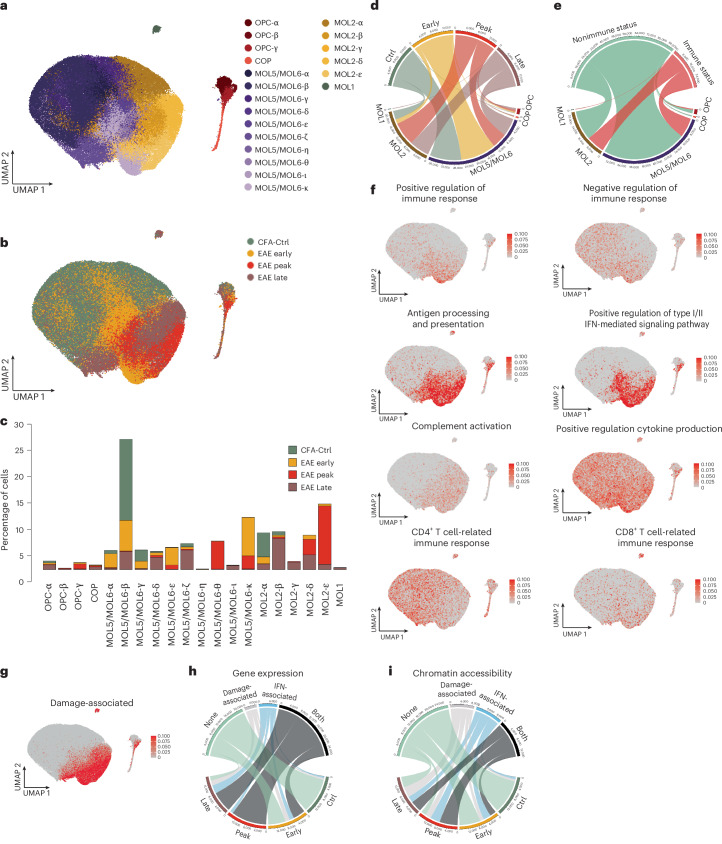
Differential immune and damage-associated transcriptional responses to EAE of MOL2 and MOL5/MOL6. **a**,**b**, Joint UMAP of OLG populations colored by cell subtypes (**a**) and time points (**b**). **c**, Bar plot of cell proportions from different time points in each OLG cell subtype. **d**, Circos plot showing the number of cells from different time points in OLG cell types (downsampled by time point). **e**, Circos plot showing the number of cells with (red) or without (green) immune status for each OLG cell type. **f**, Scaled expression level of immune response-related genes grouped by function in OLG cell subtypes. **g**, Scaled expression level of damage-associated response-related genes in OLG cell subtypes. **h**,**i**, Circos plots showing the number of cells with (black) or without (green) damage- and IFN-associated profiles, only IFN-associated profiles (blue) or only damage-associated profiles (gray) for OLG from different time points at gene expression (**h**) and chromatin accessibility (**i**) levels (downsampled by time point).

**Extended Data Fig. 2:**
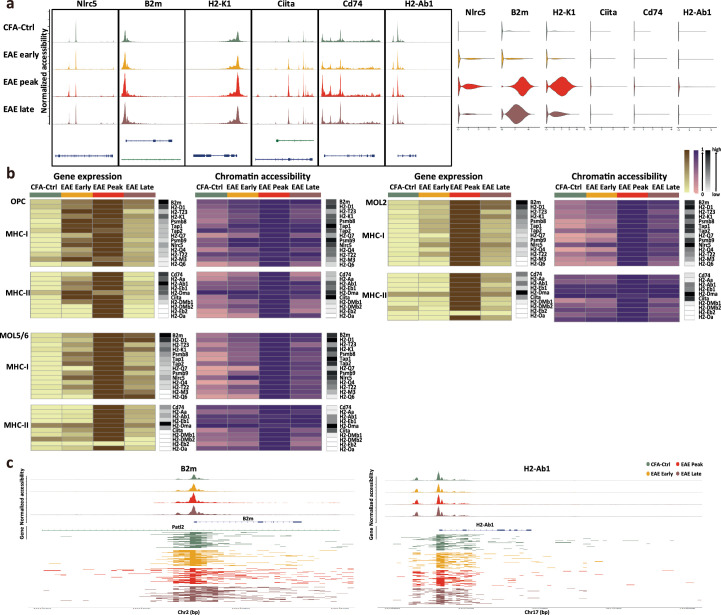
Expression and chromatin accessibility of MHC-I/II genes in different OLG cell types. **a**, Normalized chromatin accessibility (left) and log2 expression (right) of representative MHC-I and II associated genes in each time point. **b**, Heatmaps of the expression (left) and chromatin accessibility (right) of MHC-I and -II genes at different stages of EAE in OPC (left), MOL2 (upper right), and MOL5/6 (lower left). The black column on the side represents the gene raw counts. MOL1 not included due to the low number of cells. **c**, Normalized chromatin accessibility of representative MHC-I (B2m) and MHC-II (H2-Ab1) genes at different time points (top) and ATAC-seq reads alignment of 50 individual random cells per condition for the same genes (bottom).

**Extended Data Fig. 3:**
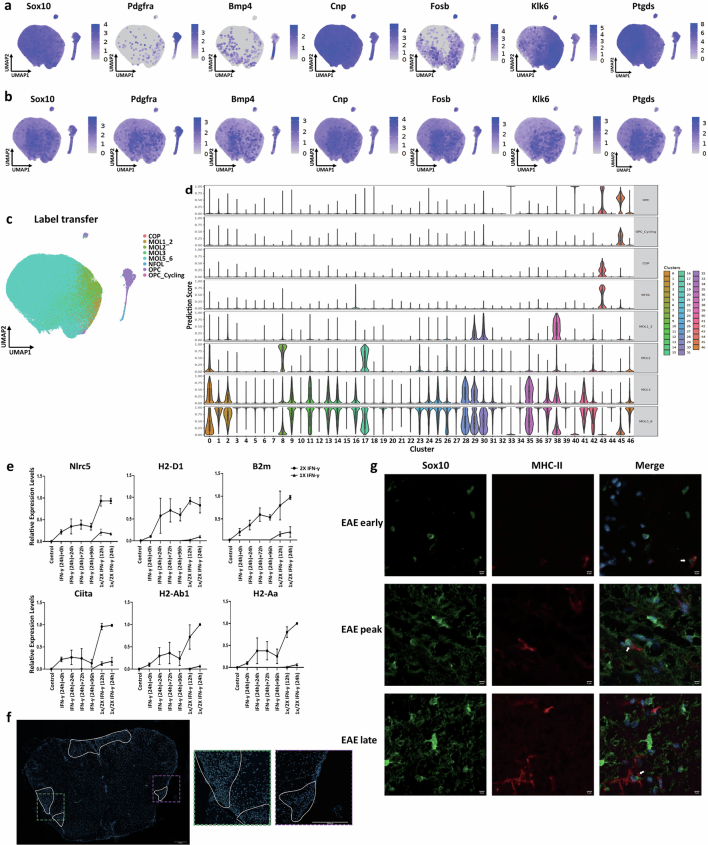
IFN-γ induces epigenetic memory in oligodendroglia and MHC2/SOX10+ cells in lesions. **a**,**b**, Feature plots showing gene expression (**a**) and gene activity score (**b**) of markers for OLG (Sox10), OPC (Pdgfra), COP (Bmp4), and MOL (Mbp, Fosb, Klk6, Ptgds) populations. **c**, UMAP of label transfer predictions from matched scRNA-seq data. **d**, Label transfer prediction scores from literature cell types to annotated clusters. 0: n = 4270, 1:3971, 2:3912, 3:3822, 4:3818, 5:3813, 6:3760, 7:3679, 8:3471, 9:3442, 10:3415, 11:3392, 12:3343, 13:3291, 14:3251, 15:3158, 16:3048, 17:2965, 18:2927, 19:2881, 20:2814, 21:2811, 22:2768, 23:2626, 24:2508, 25:2464, 26:2385, 27:2382, 28:2333, 29:2260, 30:2241, 31:2225, 32:2180, 33:2161, 34:1938, 35:1841, 36:1793, 37:1702, 38:1683, 39:1554, 40:1334, 41:1093, 42:1073, 43:1002, 44:677, 45:542, 46:164. **e**, Relative expression level (qRT-PCR) of MHC-I (upper)/II (lower) genes with 1x and 2x IFN-γ treatments in Oli-neu cells. Data are presented as mean values ± s.e.m. n = 3 biologically independent cultures per condition, with each replicate representing a separate seeding and treatment of Oli-neu cells. **f**, Representative lumbar spinal cord section from EAE peak stage with white lines outline DAPI-dense areas (lesions) (left). Zoomed-in images of the region marked by the square are shown on the right. Scale bar = 200 µm. **g**, Representative lumbar spinal cord section from EAE early, peak, and late stages, immunostained against GFP (SOX10) and immune-related protein (MHC-II). Scale bar = 5 µm. n = 3 biologically independent experiments per condition.

The expression and chromatin accessibility of some major histocompatibility complex (MHC) class I- and MHC class II-related genes were previously shown to be increased in EAE-specific OLG at peak stages of EAE^4,13^. Here, we found that, overall, the expression level of MHC class II genes was lower than that of MHC class I genes (Extended Data Fig. 2a). Notably, the expression of β-chain of MHC class I molecules (*B2m*), histocompatibility 2, K region locus 1 (*H2-K1*) and NLR family CARD domain-containing 5 (*Nlrc5*) was increased in EAE OLG at early stages (Fig. 2e and Extended Data Fig. 2a,b). We identified MOL1, MOL2 and MOL5/MOL6 mature OLG subpopulations (Fig. 3a and Extended Data Fig. 3a–d). We observed that the increase in expression of MHC class I and MHC class II genes at early stages was more expressive in OPCs than in MOL2 and MOL5/MOL6 (Extended Data Fig. 2b). The chromatin of some of the MHC class I genomic loci was accessible in OLG from CFA-Ctrl animals and exhibited a further increase in the early stages of EAE (Fig. 2e and Extended Data Fig. 2a,b). By contrast, MHC class II genes had no or low expression in OLG from CFA-Ctrl mice and showed increased expression and chromatin accessibility in OPCs at early stages of EAE, but only at the peak stage for MOLs (Fig. 2e and Extended Data Fig. 2a–c). A few MHC class I genes such as *B2m*, histocompatibility 2, D region locus 1 (*H2-D1*) and *H2-K1* remained highly expressed at late EAE stages, while the expression of most other MHC class I and MHC class II genes was notably downregulated compared to at the peak stage. Nevertheless, chromatin accessibility at the promoter/gene body of both MHC class I and MHC class II genes remained high at the late stage (Fig. 2e and Extended Data Fig. 2a–c). Thus, OLG exhibit persistent chromatin accessibility of specific immune genes at late stages of EAE. Because inflammation is decreased at these stages, this chromatin accessibility persistence in OLG might result from either epigenetic memory of peak immune-like states or from a mild inflammatory environment at late stages.

### IFNγ induces epigenetic memory in neonatal OPCs

To investigate whether a possible immune epigenetic memory in OPCs enhances immune characteristics after re-exposure to inflammatory stimuli, we treated primary mouse neonatal OPCs and the mouse OLG precursor cell line Oli-neu^18^ with IFNγ, either once or twice with a 96-h interval without IFNγ between treatments (Fig. 2f and Extended Data Fig. 3e). RNA-seq and ATAC-seq were conducted 24 h after the first or second IFNγ treatment. We observed that there was a large number of genes for which chromatin accessibility around their transcription start sites (TSSs; Supplementary Table 1) and their potential enhancers (Supplementary Table 1) was maintained or kept at a relatively higher level, in the absence of IFNγ for 96 h, after treatment with this cytokine. Many of these genes, such as *H2-Ab1*, *H2-K1* and *H2-D1*, retained chromatin accessibility in OPC populations but also in MOL populations in late-stage EAE (Fig. 2e and Extended Data Fig. 2b), suggesting epigenetic memory in this MS model. Moreover, some of these genes also exhibited a further increase in chromatin accessibility after the second IFNγ treatment (Fig. 2g,h and Supplementary Table 1). One of the possible functional outcomes of this epigenetic memory could be increased expression of these genes after a second bout of neuroinflammation. Indeed, a specific subset of the genes presenting unaltered chromatin accessibility around the TSS and/or enhancers exhibited elevated expression in OPCs after exposure to the second dose of IFNγ compared to OPCs after one dose of IFNγ treatment (Fig. 2g,h and Supplementary Table 1). Moreover, in the Oli-neu cell line, quantitative PCR with reverse transcription (RT–qPCR) analysis revealed increased expression of specific genes within the MHC pathway, such as *Nlcr5*, *H2-D1*, *H2-Ab1*, *H2-Aa*, *B2m* and class II MHC transactivator (*Ciita*), when comparing the two IFNγ treatments to a single IFNγ treatment (Extended Data Fig. 3e). These results from cultured neonatal OPCs suggest that OPCs might retain, at the level of chromatin accessibility, epigenetic memory of a previous neuroinflammatory insult, which might make them more prone to transcriptionally reactivate an immunological profile. However, additional studies are needed to determine whether a similar epigenetic memory phenomenon occurs in adult OPCs and MOLs, given their distinct transcriptomic and epigenomic profiles.

### RNAscope confirms imOLG across EAE stages

Our multiome data suggested that imOLG are present not only at the peak stage but also at the early and late stages of EAE. We thus used RNAscope in situ hybridization (ISH) to understand where these imOLG were located relative to EAE lesions. We defined lesions as white matter regions with a high number of cells due to inflammatory infiltrates (Extended Data Fig. 3f). Consistent with the multiome results, the MHC class II^+^ OLG (*Sox10*^+^*H2-Ab1*^+^) were observed in EAE spinal cord sections from the peak stage but also in early and late stages, albeit in different proportions (Fig. 2i,j and quantification in the Methods). There was no significant difference in the percentages of imOLG between lesion and nonlesion areas for all three stages (Fig. 2j; *P* > 0.05). Consistent with previous observations^4^, we confirmed the presence of imOLG at the protein level by immunostaining against MHC class II (I-A/I-E), along with SOX10 (GFP; Extended Data Fig. 3g). Together, our data indicate that MHC class I and MHC class II genes maintain latent transcriptional and epigenetic states in OLG at late EAE stages, which may result in immune gene expression when facing recurrent inflammatory stimuli and contribute to the chronic persistent disease state.

### Stronger immune transcriptional responsiveness of MOL2

**Extended Data Fig. 4:**
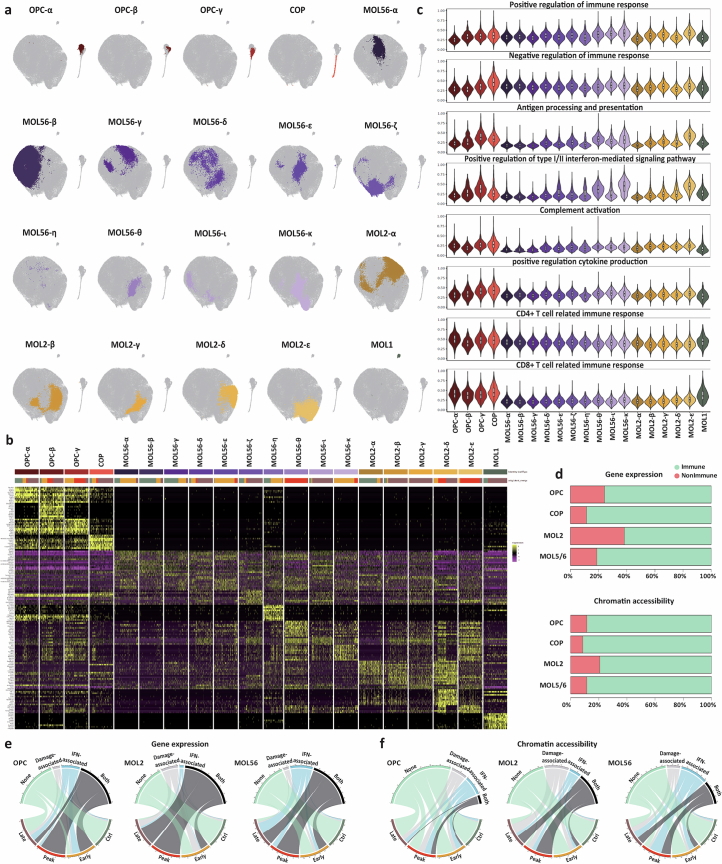
MOL sub-cell type classification based on gene expression and their IFN-associated and damage-associated transcriptional responses. **a**, Joint UMAP of OLG populations colored by sub-cell types, on top of UMAP with all cells (in gray). **b**, Heatmap of the scaled expression of differentially expressed genes between OLG sub-cell types (percentage of cells in a cluster expressing a gene superior to 50%, log2FC > 0.5 and adjusted p-value < 0.05), colored by sub-cell types and time points. **c**, Scaled expression level of immune response related genes grouped by function in OLG sub-cell types. OPC-α:n = 2134, OPC-β:289, OPC-γ:1072, COP:1002, MOL56-α:4706,MOL56-β:37969, MOL56-γ:5595, MOL56-δ:4166, MOL56-ε:4733, MOL56-ζ:6299, MOL56-η:164, MOL56-θ:3392, MOL56-ι:1093, MOL56-κ:10704, MOL2-α:10854, MOL2-β:8988, MOL2-γ:1841, MOL2-δ:6227, MOL2-ε:8413, MOL1:542. **d**, Percentage of cells with (red) or without (green) immune status identified by gene expression (left) and chromatin accessibility (right) in each OLG sub-cell type. **e**,**f**, Circos plot showing the number of cells with (black) or without (green) damage- and IFN-associated profiles, only IFN-associated profile (blue), only damage-associated profiles (gray) for OPC (left), MOL2 (middle), and MOL5/6 (right) from different time points at gene expression (**e**) and chromatin accessibility (**f**) levels (downsampled by time point).

MOLs have recently been shown to be heterogeneous, with specific populations exhibiting regional preferences and different susceptibility to spinal cord injury^4,7,13,14,19,20^. Based on distinct gene expression profiles within OLG, we could further subdivide OPCs into three (OPC-α–OPC-γ), MOL2 into five (MOL2-α–MOL2-ε) and MOL5/MOL6 into ten cell states/subpopulations (MOL5/MOL6-α–MOL5/MOL6-κ; Fig. 3a and Extended Data Fig. 4a). Interestingly, most of these states display prevalence toward specific EAE time points (Fig. 3a–c) and present specific expression of gene modules (Methods, Extended Data Fig. 4b and Supplementary Table 2). Overall, we observed a higher proportion of MOL2 at the peak and late stages of EAE than CFA-Ctrl and early-stage EAE (Fig. 3b–d). This increase could arise from conversion of MOL5/MOL6 into MOL2, being more resilient to the neuroinflammatory environment, and/or preferential differentiation of OPCs into MOL2 in the context of EAE. We then investigated whether any of these MOL populations were more prone to transition into immune-like states in EAE. In total, 38.35% of MOL2 were identified as imOLG, whereas only 18.75% of MOL5/MOL6 underwent this transition (Fig. 3e and Extended Data Fig. 4d), indicating that MOL2 has higher immune responsiveness. Within the identified immune-related genes, some were more enriched in specific MOL populations, whereas others, involved, for instance, in cytokine and T cell immune responses, showed similar expression levels among all MOLs (Fig. 3f and Extended Data Fig. 4c). Overall, our results suggest that MOL2 exhibit a stronger immune response to the neuroinflammatory environment than MOL5/MOL6 and may play a more important role in disease progress.

### Damage-associated transcriptional responses in OLG

In addition to the immune response, the disease-associated profile of OLG can also include a damage-associated response, which is related to apoptosis and survival mechanisms^8,21^. We found that the majority of OLG at the peak stage expressed high levels of both damage- and IFN-associated genes, whereas a subset of cells from the early and late stages expressed either damage- or IFN-associated profiles (Fig. 3g,h). By contrast, chromatin accessibility showed a stronger specificity in either damage- or IFN-associated genes (Fig. 3i), suggesting that the chromatin accessibility of these IFN- or damage-associated genes was already reduced at the peak stage, despite their transcription. A higher percentage of MOL2 displayed both damage- and IFN-associated profiles than MOL5/MOL6 at peak and late stages (Extended Data Fig. 4e). At the late stage, a greater proportion of MOL2 exhibited a damage-associated profile, whereas MOL5/MOL6 showed a higher prevalence of IFN-associated profiles. Additionally, we identified a subset of OPCs at the late stage that did not express either damage- or IFN-associated profiles (Fig. 3f,g), suggesting that these OPCs may have a nondisease phenotype and could potentially contribute to remyelination at the chronic stage of disease. Similar to immune-related genes, fewer damage- and IFN-associated OLG were identified by chromatin accessibility than gene expression (Fig. 3h,i and Extended Data Fig. 4e,f).

### Distinct transcriptional response of MOL2 and MOL5/MOL6 in EAE

**Fig. 4:**
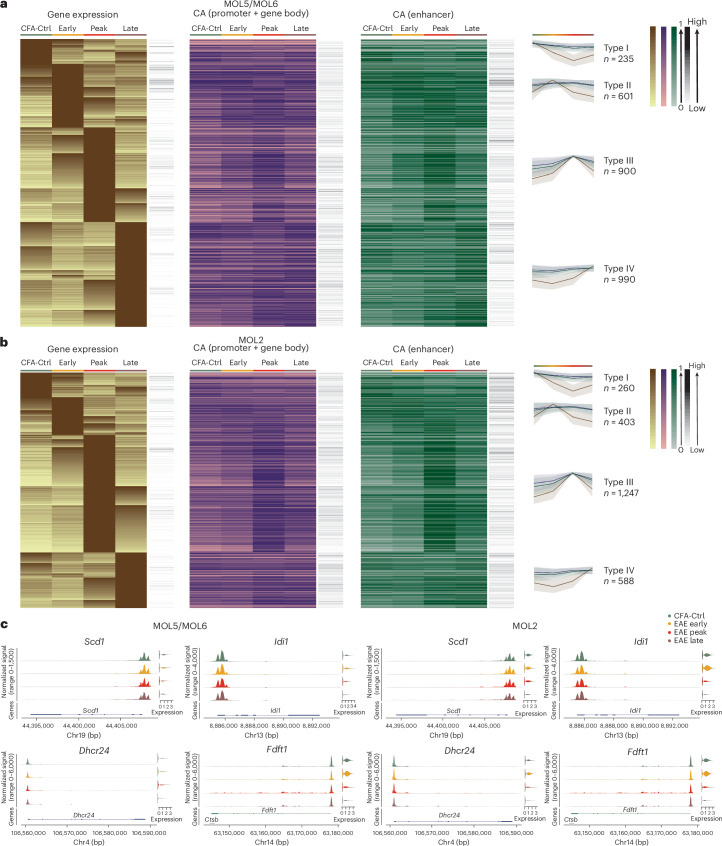
Increase of cholesterol biosynthetic processes in MOL5/MOL6 at early stages of EAE. **a**,**b**, Heat maps of differentially expressed genes (DESeq2 Wald test and Benjamini–Hochberg multiple testing correction with a log_2 _(fold change) (log_2_ (FC)) of >1 and adjusted *P* value of <0.01; brown) between different time points in MOL5/MOL6 (**a**) and MOL2 (**b**), chromatin accessibility at promoters and gene bodies (purple) and chromatin accessibility at enhancer regions (green) of the same gene. The black column on the right represents the raw gene counts. The line plots represent the mean of the normalized and scaled gene expression (line in brown), chromatin accessibility at the promoter and gene bodies (line in purple) and chromatin accessibility at enhancer regions (line in green) of genes in different groups. The color band associated with a line represents the standard deviation of the mean. Differentially expressed genes and associated GOs are shown in Supplementary Table 3 and 4. **c**, Normalized chromatin accessibility (left of each gene panel) and log_2_ expression (right of each gene panel) of genes related to the cholesterol biosynthetic process (*Scd1*, *Idi1*, *Dhcr24* and *Fdft1*) in MOL5/MOL6 (left) and MOL2 (right).

**Extended Data Fig. 5:**
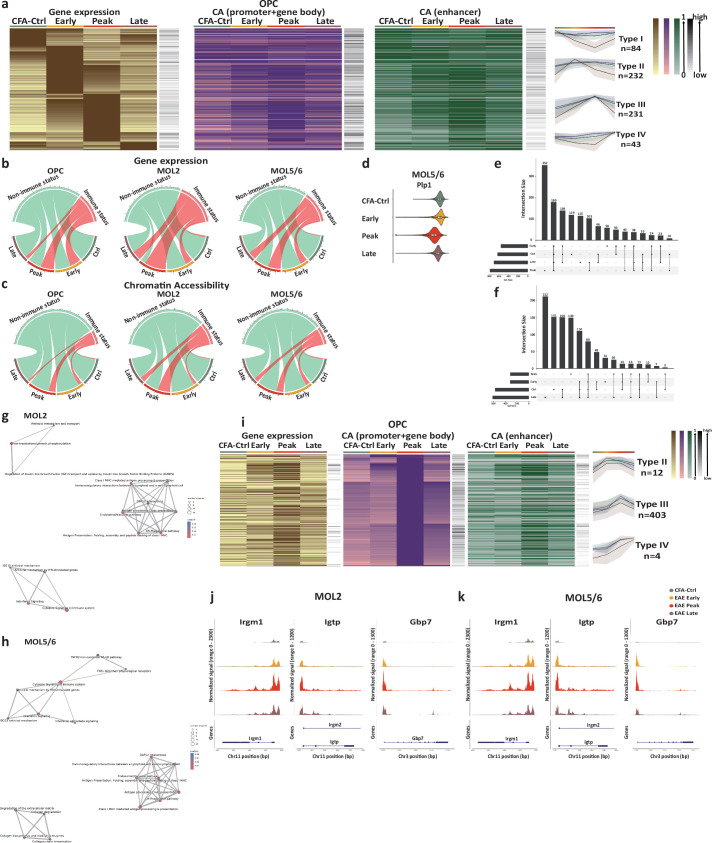
Immune-related genes showed substantial changes in expression and chromatin accessibility, with increased transcription in OPCs and MOL2 at early and peak EAE stages. **a** Heatmaps of differentially expressed genes (log2FC > 1 and p-value adjusted < 0.01) (in brown) between different time points in OPC, with chromatin accessibility at promoter and gene body (in purple) and at enhancer regions (in green) of the same gene. The black column on the side represents the gene raw counts. The line plots represent the averages of gene expression (line in brown), the chromatin accessibility at promoter and genes body (line in purple), and chromatin accessibility at enhancer regions (line in green) of genes in different groups. The color band associated with a line represents the standard error of the distribution. **b**,**c**, Circos plots showing the number of cells with (red) or without (green) immune status identified with gene expression (**b**) and chromatin accessibility (**c**) from different time points in OPC (left), MOL2 (middle) and MOL56 (right) (downsampled by time points). **d**, Violin plots showing the expression of myelination related gene Plp1 at different stages in MOL5/6. n=CFA-Ctrl:43293,Early:30937,Peak:15248,Late:30705. **e**, **f** Upset plots showing differentially expressed genes overlaps between MOL2 over MOL5/6 (**e**) or MOL5/6 over MOL2 (**f**), within each time point (Log2 Fold Change > 1 and q-value < 0.01)). **g**,**h**, Connections between enriched GO terms (p.adjust < 0.05) of genes with both differential expression and chromatin accessibility among different disease stages in MOL2 (**g**) and MOL5/6 (**h**). **i**, Heatmaps of differential chromatin accessibility at promoter and gene body (log2FC > 1 and p-value adjusted < 0.01) (in purple) between different time points in OPCs, of gene expression (in brown) and chromatin accessibility at enhancer regions (in green) of the same gene. Other features are such as in **a**).**j**,**k**, Chromatin accessibility normalized tracks of representative immune related genes (Irgm1, Igtp and Gbp7) with an important increase at peak stage in MOL2 (**j**) and MOL5/6 (**k**).

To explore whether other biological processes were dynamically modulated in OLG during the course of EAE, we performed differential gene expression analysis across the different stages in different OLG cell types (Methods). The differentially expressed genes were divided into Type 1 (high expression in CFA-Ctrl), Type 2 (high expression at the early stage), Type 3 (high expression at the peak stage) and Type IV (high expression at the late stage) main types and also subtypes (high expression in a main type associated with high expression in a secondary type; Fig. 4a,b and Extended Data Fig. 5a).

In OPCs, we observed an increase in IFN and cytokine signaling at early and peak stages (secondary subtype division Type 2–Type 3), and, as expected, genes related to antigen processing cross-presentation were increased in expression at the peak stage (Type 3), followed by a notable reduction at the late stage (Extended Data Fig. 5a and Supplementary Tables 3 and 4). Furthermore, genes related to neuronal system and development, albeit not significant in Gene Ontology (GO) terms, such as doublecortin (*Dcx*) and glutamate receptor-interacting protein 1 (*Grip1*), were upregulated at the late stage (Type 4) in OPCs (Extended Data Fig. 5a and Supplementary Tables 3 and 4). OPCs showed a drastic reduction of immune-associated cells at the gene expression level and, to some extent, at the chromatin level at the late stage of EAE compared to all other time points and MOL populations (Extended Data Fig. 5b,c). However, many of these genes retained chromatin accessibility, suggesting that they retain the epigenetic potential to initiate their transcription.

Consistent with OPCs, the differentially expressed genes of MOL5/MOL6 were also divided into four distinct types and subtypes (Fig. 4a). Most of the genes with high expression at the peak stage (Type 3) in MOL5/MOL6 were immune related, such as antigen processing/cross-presentation (*Psmb8*, *Psmb9* and *Tap1*), but their expression decreased drastically at the late stage (Fig. 4a and Supplementary Tables 3 and 4). We observed an increase of interleukin signaling genes in MOL5/MOL6 (Type 2) at the early stage (Fig. 4a and Supplementary Tables 3 and 4). We found an increase in cholesterol biosynthetic process-related genes at early stages (Type 2 subtype 1), such as stearoyl-CoA desaturase-1 (*Scd1*), isopentenyl-diphosphate-δ isomerase 1 (*Idi1*), 24-dehydrocholesterol reductase (*Dhcr24*), farnesyl-diphosphate farnesyltransferase 1 (*Fdft1*), squalene epoxidase (*Sqle*), farnesyl-diphosphate synthase (*Fdps*) and methylsterol monooxygenase 1 (*Msmo1*) in MOL5/MOL6. This increase in cholesterol-related pathway genes was also found in early stages (Type 2 subtype 1) in OPCs (Fig. 4a,c and Supplementary Tables 3 and 4). The formation of the myelin sheath necessitates highly coordinated levels of fatty acid and lipid synthesis process-related genes^22^. MOLs have been recently shown to be able to contribute in some extent to remyelination^23–26^. The increase in expression of cholesterol biosynthesis-related genes at the initial stage could suggest a form of preliminary remyelination adaptation to the very first cues given by EAE-driven demyelination (Extended Data Fig. 5d). At the late stage (Type 4), we observed the increased expression of genes in MOL5/MOL6 involved in extracellular matrix degradation and collagen chain trimerization, suggesting simultaneous matrix degradation and synthesis for tissue repair during chronic phases of the disease. We also observed increased expression of genes involved in the RHO GTPase cycle in MOL5/MOL6 at a secondary subtype division at late and early stages (Type 4 subtype 2), such as STEAP3 metalloreductase (*Steap3*) and copine 8 (*Cpne8*; Supplementary Tables 3 and 4) involved in actin cytoskeleton and microtubule processes, which play a role in myelination^27^, further suggesting that MOL5/MOL6 might activate gene regulatory programs associated with regeneration during these stages.

We also identified four groups and subgroups of genes with differential expression between different time points for MOL2 (Fig. 4b). Similar to MOL5/MOL6, we found that cholesterol biosynthetic process-related genes also had a transitory increase in MOL2 at early-stage EAE compared to later stages (Fig. 4b,c and Supplementary Tables 3 and 4). Many genes with high expression in MOL2 at the peak stage were immune-related genes (Type 3, such as *Tap1*, histocompatibility 2, T region locus 23 (*H2-T23*), integrin subunit-α 9 (*Itga9*) and *Psmb8*, which is consistent with the exacerbated immune response and higher number of imOLG at the peak stage (Fig. 4b and Supplementary Tables 3 and 4). Interestingly, we also found that genes related to development and axon guidance, different from Type 1 genes, exhibited high expression in MOL2 at the late stage, such as plexin A4 (*Plxna4*), but also semaphorin 3B (*Sema3b*), EPH receptor (*Ephb2* and *Epha10*) and ankyrin 1 (*Ank1*; Fig. 4b and Supplementary Tables 3 and 4). In summary, our multiome data indicate that MOL2 initiate, as MOL5/MOL6, a cholesterol biosynthesis program in response to the arising neuroinflammatory environment but transition to an immune-like state during the course of EAE.

We then explored expression differences between MOL2 and MOL5/MOL6 at each time point (Methods and Supplementary Table 5). At the peak stage of EAE, we identified 352 genes with increased expression in MOL2 compared to MOL5/MOL6 (Extended Data Fig. 5e), many of which were associated with immune responses and apoptotic processes, including *Ciita*, *Gbp5*, *Jun* and death-associated protein (*Dap*; Supplementary Tables 5 and 6). This finding supports that MOL2 exhibit a stronger immune profile than MOL5/MOL6. For MOL5/MOL6, we observed 80 genes with increased expression across different time points (Extended Data Fig. 5f). Among these, we identified several genes related to the neuronal system but also cell development, such as meis homeobox 2 (*Meis2*), neuroligin 3 (*Nlgn3*) and *Sema6a*, at early stages (Supplementary Tables 5 and 6).

### Increased MOL chromatin accessibility at immune genes in EAE

**Fig. 5:**
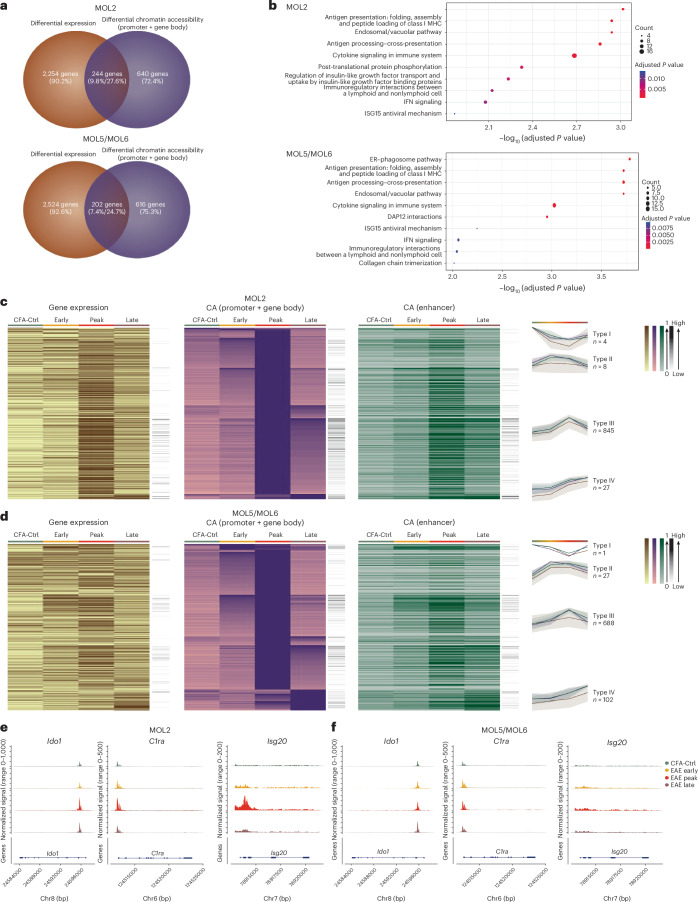
Stronger epigenetic immune response at the chromatin accessibility level in MOL2 than in MOL5/MOL6. **a**, Venn diagram showing the number of genes with differential expression and/or differential chromatin accessibility among different disease stages (DESeq2 Wald test and Benjamini–Hochberg multiple testing correction with a log_2 _(FC) of >1 and adjusted *P* value of <0.01) in MOL2 (top) and MOL5/MOL6 (bottom). **b**, Top GO pathways by biological process terms (Fisher exact test and multiple testing correction using FDR with an adjusted *P* value of <0.05) for genes with both differential expression and differential chromatin accessibility in MOL2 (top) and MOL5/MOL6 (bottom) among different disease stages. **c**,**d**, Heat maps of differential chromatin accessibility at promoters and gene bodies (DESeq2 Wald test and Benjamini–Hochberg multiple testing correction with a log_2 _(FC) of >1 and adjusted *P* value of <0.01; purple) between different time points in MOL2 (**c**) and MOL5/MOL6 (**d**), gene expression (brown) and chromatin accessibility at enhancer regions (green) of the same gene. The black column on the right represents the gene raw counts. The line plots represent the average gene expression (line in brown), chromatin accessibility at promoters and gene bodies (line in purple) and chromatin accessibility at enhancer regions (line in green) of genes in different groups. The color band associated with a line represents the standard deviation of the mean. Differential chromatin accessibility at promoters/gene bodies and associated GOs are shown in Supplementary Tables 3 and 4. **e**,**f**, Normalized chromatin accessibility of immune system process-related genes (*Ido1, C1ra* and *Isg20*) in MOL2 (**e**) and MOL5/MOL6 (**f**) at each time point.

Differences between MOL2 and MOL5/MOL6 at the transcriptional level might correlate with changes at the epigenetic level. We thus compared the list of genes with differential chromatin accessibility at promoter/gene body regions and genes with differential expression across different stages and found that multiple genes showed notable changes in both expression and chromatin accessibility (Fig. 5a and Supplementary Table 7). Most were immune related (Fig. 5b, Extended Data Fig. 5g,h and Supplementary Tables 7 and 8). This indicated that immune-related genes tend to have important changes in both gene expression and chromatin accessibility at promoter/gene body regions in all OLG populations.

We then explored the dynamics of chromatin accessibility both at promoters/gene bodies and at associated distant enhancer regulatory regions during the disease course. Gene loci displaying differential chromatin accessibility at promoter/gene body regions between different time points were divided into high chromatin accessibility in CFA-Ctrl (Type 1), early stage (Type 2), peak stage (Type 3) and late stage (Type 4; Fig. 5c,d). Very few genes could be found in Type 1 in MOLs and none could be found in OPCs (Fig. 5c,d and Extended Data Fig. 5i). In both OPCs and MOLs, most genes with differential chromatin and enhancer accessibility reached their highest levels of accessibility during the peak stage, with the association of immune-related functions (Supplementary Tables 3 and 4). Of note, a predominance in accessibility at genes associated with collagen biosynthesis and extracellular matrix organization was described in MOL5/MOL6 compared to MOL2 at late stages of EAE (Fig. 5c,d and Supplementary Tables 3 and 4).

When comparing MOL2 to MOL5/MOL6, we found that there was a group of antigen processing/presentation with high chromatin accessibility at the peak stage in both MOL2 and MOL5/6 (Extended Data Fig. 5j,k and Supplementary Tables 3 and 4). Moreover, we found that more immune-related genes were upregulated in MOL2 than in MOL5/MOL6 at the peak stage. Some immune system process-related genes, such as indoleamine 2,3-dioxygenase 1 (*Ido1*), complement component 1, r subcomponent A (*C1ra*) and IFN-stimulated exonuclease gene 20 (*Isg20*) showed increased chromatin accessibility at the peak stage in MOL2 but not in MOL5/MOL6 (Fig. 5e,f and Supplementary Tables 3 and 4), further indicating that MOL2 triggers a more robust opening of the chromatin at immune genes after EAE than MOL5/MOL6.

### HOXB enhancer activity increases in MOL5/MOL6 in late EAE

**Fig. 6:**
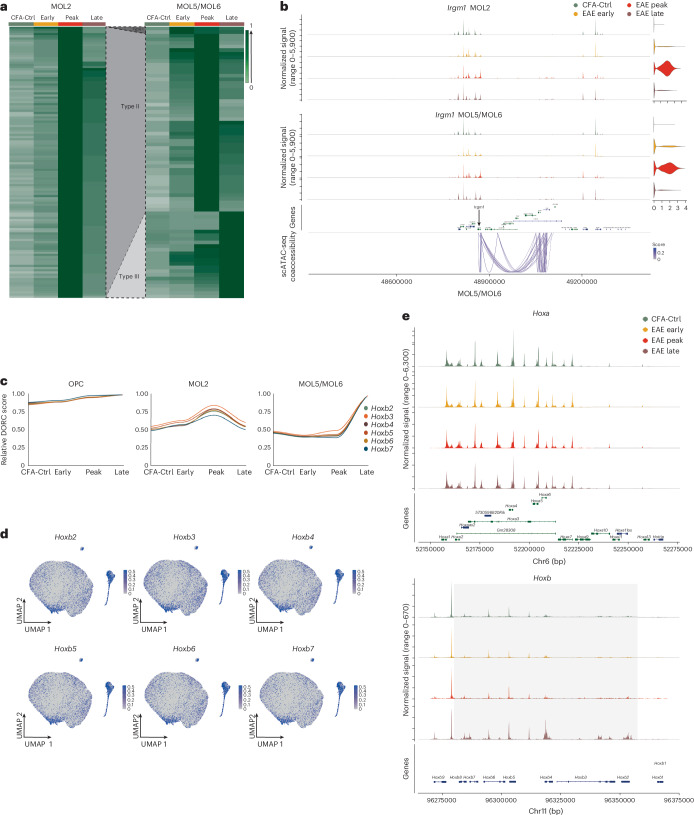
Increased chromatin accessibility at DORCs at the *Hoxb* locus in MOL5/MOL6 at late-stage EAE. **a**, Heat maps of normalized and scaled DORC scores of differentially expressed genes (DESeq2 Wald test and Benjamini–Hochberg multiple testing correction with a log_2_ (FC) of >1 and adjusted *P* value of <0.01) at different stages, 124 genes in MOL2 and 75 genes in MOL5/MOL6. DORCs were classified into three types: Type 1 with high activity in early-stage EAE, Type 2 with increased activity at the peak stage and Type 3 with increased activity at the late stage. Differential DORCs and associated GOs are shown in Supplementary Tables 3 and 4. **b**, Representative gene (*Irgm1*) in Type 2 DORCs in MOL2 (top) and MOL5/MOL6 (bottom). The genomic track represents its accessibility at different time points, and the links denote the significant correlation (*P* < 0.05) between peaks and *Irgm1* (±500 kb from TSSs). The violin plots show *Irgm1* expression in MOL2 (top) and MOL5/MOL6 (bottom). **c**, Normalized and scaled DORC score for *Hoxb* cluster genes (Type 3 DORCs in MOL5/MOL6) in OPC (left), MOL2 (middle) and MOL5/MOL6 (right). **d**, Feature plots showing DORC scores of *Hoxb2*, *Hoxb3*, *Hoxb4*, *Hoxb5*, *Hoxb6* and *Hoxb7*. UMAP coordinates and population distributions are as in Fig. 3a. **e**, Normalized chromatin accessibility of *Hoxa* (top) and *Hoxb* (bottom) genes in MOL5/MOL6; the gray box highlights *Hoxb* genes in **d**.

**Extended Data Fig. 6:**
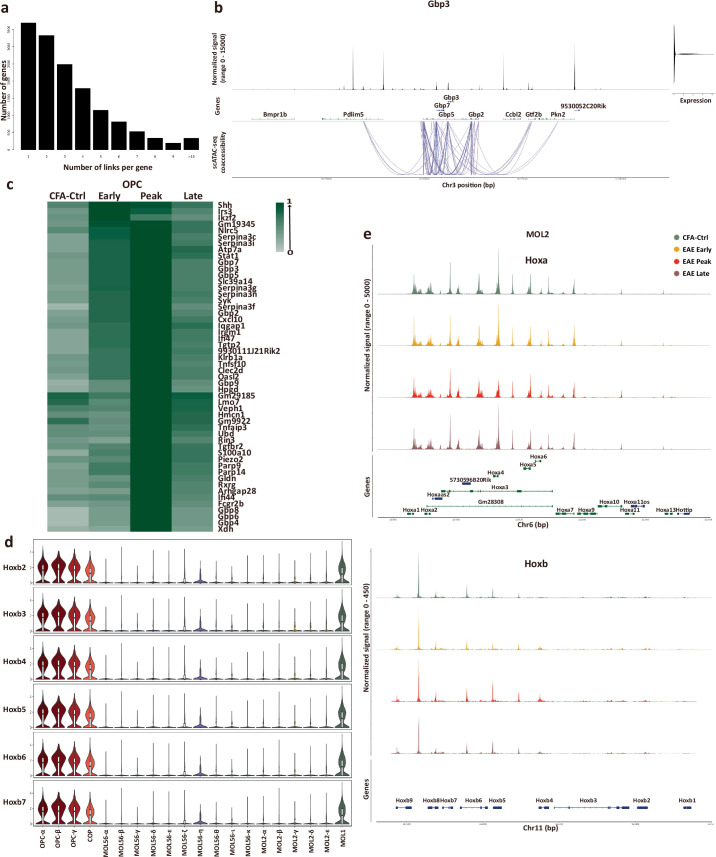
Priming DORCs of immune related genes in MOL2 at early and peak stages and MOL5/6 at peak stage. **a**, Bar plot of the number of significant peak-gene associations. **b**, Representative DORC (Gbp3). The genomic track represents the OLG bulk accessibility of Gbp3 while the links denote the significant correlation (p-value < 0.05) between peaks and Gbp3 (± 500 kb from TSSs). The violin plot shows Gbp3 log2 expression in OLG. **c**, Heatmaps of normalized and scaled DORC score of differentially variable gene scores (log2FC > 1 and p-value adjusted < 0.01) at different stages, 52 genes in OPC. Differential DORCs and associated gene ontologies are shown in Supplementary Table 3, 4. **d**, Violin plots of DORCs score of Hoxb genes in OLG sub-cell types. OPC-α: n = 2134, OPC-β:289, OPC-γ:1072, COP:1002, MOL56-α:4706, MOL56-β:37969, MOL56-γ:5595, MOL56-δ:4166, MOL56-ε:4733, MOL56-ζ:6299, MOL56-η:164, MOL56-θ:3392, MOL56-ι:1093, MOL56-κ:10704, MOL2-α:10854, MOL2-β:8988, MOL2-γ:1841, MOL2-δ:6227, MOL2-ε:8413, MOL1:542. **e**, Normalized chromatin accessibility of Hoxa (upper) and Hoxb (lower) genes in MOL2.

Recently, superenhancers, clusters with high levels of transcription factor binding and domains of regulatory chromatin (DORCs), have been suggested to have key roles in modulating gene expression^12^. Genes associated with DORCs were characterized by exceptionally large (five or more) numbers of significant peak–gene associations (Extended Data Fig. 6a,b). To explore the role of DORCs in the course of EAE, we defined differentially accessible domain-regulated genes in OLG subpopulations (Fig. 6a and Extended Data Fig. 6c). We found three types of DORCs in MOL5/MOL6, among which only 2 domains were classified as Type 1 (high activity at the early stage), 49 domains were classified as Type 2 (high activity at the peak stage), and 24 domains were classified as Type 3 (high activity at the late stage; Fig. 6a). By contrast, we only found Type 2 (high activity at the peak stage) DORCs in MOL2. Type 2 DORCs in both MOL2 and MOL5/MOL6 were associated with genes involved in immune processes, with *Gbp7*, IFN regulatory factor 4 (*Irf4*) and *Irgm1* included (Fig. 6a,b and Supplementary Table 3). For OPCs, 3 Type 1 DORCs and 49 Type 2 DORCs were identified, with many of them being immune related (Fig. 6b and Supplementary Table 3).

The presence of two additional DORC types (Types 1 and 3) in MOL5/MOL6 suggests that additional biological processes are regulated in this mature OL subtype compared to MOL2 (Fig. 6c). In particular, at this stage, we found increased DORCs regulating a group of homeobox (*Hoxb*) genes, such as *Hoxb2*, *Hoxb3* and *Hoxb4*, in MOL5/MOL6 but not MOL2 (Fig. 6c–e, Extended Data Fig. 6d,e and Supplementary Table 3). Interestingly, only a subset of MOL5/MOL6, a subpopulation of MOL5/MOL6-ζ, mainly from late-stage EAE, was driving the upregulation of DORCs of *Hoxb* genes (Fig. 6d). By contrast, all subtypes of OPCs present high DORC scores across all time points (Fig. 6c and Extended Data Fig. 6d). The increased activity was only found in *Hoxb* but not in other HOX family gene regions (Fig. 6e and Extended Data Fig. 6e). HOXB2 is essential for OL patterning^28^. Thus, the increased chromatin accessibility of these developmental-related DORCs suggests that MOL5/MOL6 might have primed transcriptional programs compatible with nervous system repair and remyelination and promotion during the late stages of EAE.

### MultiVelo reveals distinct MOL2 and MOL5/MOL6 responses in EAE

**Fig. 7:**
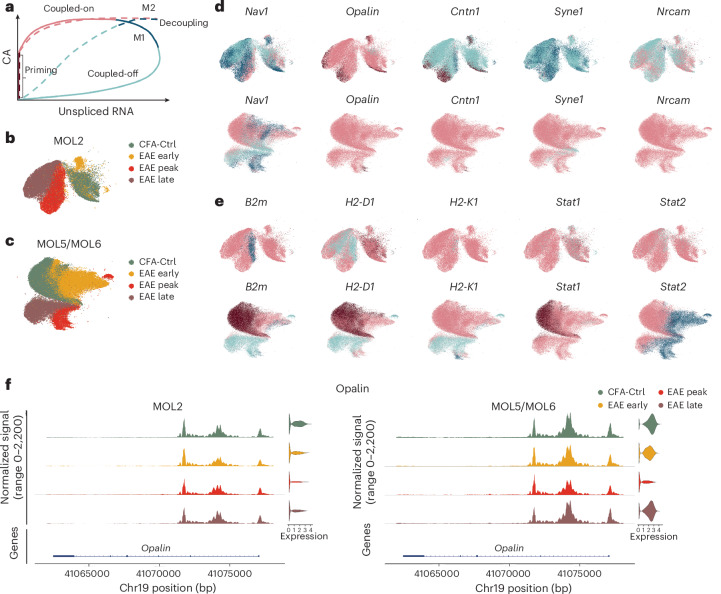
MultiVelo analysis indicates divergent responses of MOL2 and MOL5/MOL6 cells to the evolving disease environment in EAE. **a**, Gene phase portraits predicted by MultiVelo for model 1 (M1, full line) and model 2 (M2, dotted line) genes. **b**,**c**, MultiVelo UMAPs of MOL2 (**b**) and MOL5/MOL6 (**c**) cells colored by time points. **d**,**e**, MultiVelo UMAPs of MOL2 and MOL5/MOL6 cell genes colored by gene state assigned by MultiVelo for representative central nervous system development- (**d**) and immune response-related (**e**) genes. *Nav1*, *Opalin*, *Cntn1*, *Syne1*, *Nrcam*, *Stat1* and *Stat2* are classified as M1 genes, and *B2m*, *H2-D1* and *H2-K1* were classified as M2 genes. **f**, Normalized chromatin accessibility (left) and log_2_ expression (right) of *Opalin* in MOL2 and MOL5/MOL6 cells.

**Extended Data Fig. 7:**
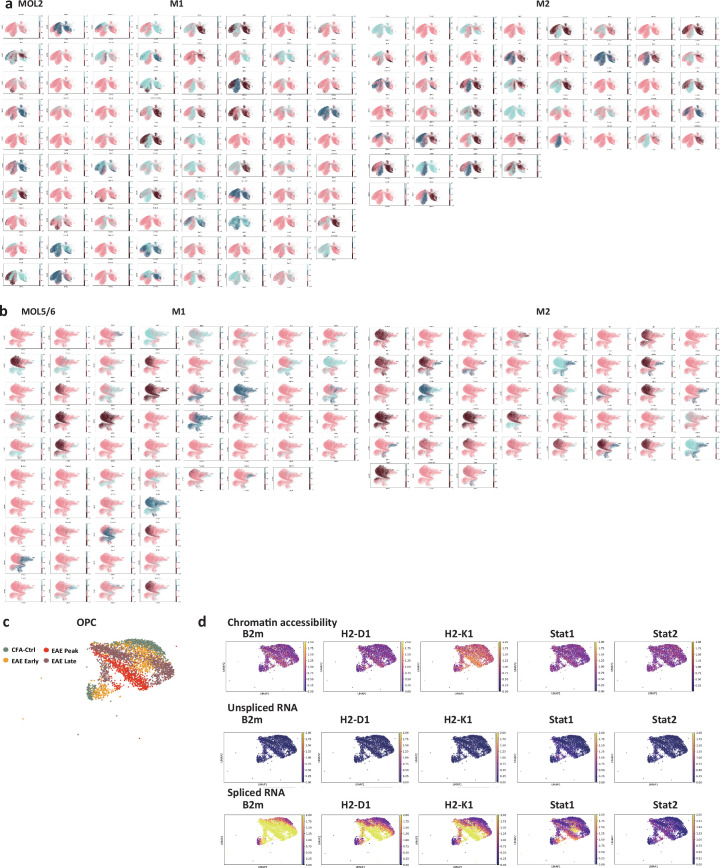
MultiVelo analysis classified genes into two models. **a**,**b**, UMAPs of MOL2 (**a**) and MOL5/6 (**b**) colored by gene state assigned by MultiVelo, model 1 (left) (M1, chromatin is closing before transcriptional repression) and model 2 (right) (M2, chromatin is closing after transcriptional repression). **c**, MultiVelo UMAP of OPC colored by time points. **d**, Depiction of chromatin accessibility (top), moments of unspliced RNA (middle) and spliced RNA (bottom), calculated by MultiVelo for B2m, H2-D1, H2-K1, Stat1 and Stat2 in OPC.

Although RNA velocity leverages splicing and RNA turnover to infer cellular transitional dynamics^29^, another key component for these dynamics are changes in the epigenomic landscape during cell transitions. To explore dynamics of OLG during disease, we applied MultiVelo^30^, a tool that integrates transcriptomics and epigenomics datasets to estimate cell-fate predictions. For a given gene, this tool can define the state of each cell into one of the following four phases: priming (brown), coupled-on (pink), decoupling (dark blue) and coupled-off (light blue; Methods, Fig. 7a and Extended Data Fig. 7a,b)^30^. Due to the limited number of OPCs, some genes of interest did not present a complete transcriptional trajectory using MultiVelo (Methods and Extended Data Fig. 7c,d). We found that canonical OLG genes, such as *Opalin*, contactin 1 (*Cntn1*) and neuron navigator 1 (*Nav1*), showed coupled-on phases in MOL5/MOL6 in both CFA-Ctrl mice and in mice with EAE from early to late stages (Fig. 7c,d,f). However, many of these nervous system development-related genes exhibited either a priming or coupled-off phase in MOL2 (Fig. 7b,d,f). Thus, similar to our previous results, Multivelo analysis suggests a differential regenerative response of MOL2 and MOL5/MOL6 in the context of EAE. By contrast, immune-related genes, such as *B2m*, *H2-D1* and *Stat1*, were observed to transition between the coupled-on and decoupling phases in most MOL2 cells, which indicated a highly open chromatin level and transcription (Fig. 7b,e). However, these immune genes showed a chromatin priming phase in MOL5/MOL6 in CFA-Ctrl animals, followed by a transient coupled-on phase at early-stage EAE. At the peak and late stages, *B2m* and *H2-D1* transitioned into a coupled-off phase, indicating an important reduction in chromatin accessibility and gene expression of these immune-related genes at the chronic stage of the disease (Fig. 7c,e). Transition to an immune-like state has been previously shown to be incompatible with differentiation in OPCs^5^, and our data suggest that in MOL in EAE the enhanced immune-like state of MOL2 cells is distinct to a putative more remyelination-prone state of MOL5/MOL6 cells. Thus, these results further indicate a divergence in the response of different MOL populations to the evolving disease environment in EAE.

### Changes in MOL transcription factor activity in EAE

**Fig. 8:**
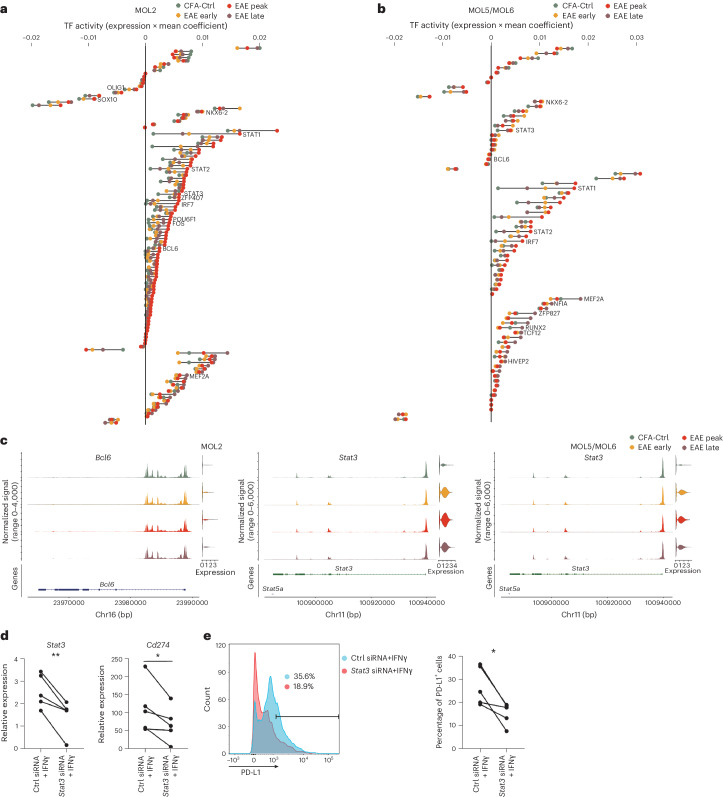
Transcription factor activity foresees the immunosuppression potential of MOLs. **a**,**b**, Ranked transcription factor activities of the predicted transcription factors at different stages in MOL2 (**a**) and MOL5/MOL6 (**b**). The *x* axis shows transcription factor activity represented with a dot colored by stage and connected with a line. Transcription factor activity was calculated as the mean coefficient multiplied by the average expression in each time point. The *y* axis shows transcription factors ranked based on transcription factor activity at each time point (a zoom-in view is shown in Extended Data Fig. 9); TF, transcription factor. **c**, Normalized chromatin accessibility (left) and log_2_ expression (right) of *Bcl6* and *Stat3* in MOL2 (left) and *Stat3* in MOL5/MOL6 (right). **d**, Relative expression of *Stat3* and *Cd274* in OPCs treated with control siRNA or *Stat3* siRNA after IFNγ treatment, measured by RT–qPCR; **P < 0.01 and *P < 0.05. Student’s two-tailed paired *t*-tests were used for comparisons between matched conditions (*P* = 0.0098 for *Stat3* and *P* = 0.0409 for *Cd274*); *n* = 5 biologically independent samples per condition, each derived from primary OPC cultures isolated from a different mouse. **e**, Percentages of PD-L1^+^ OPCs treated with control siRNA or *Stat3* siRNA after IFNγ treatment measured by flow cytometry; *P < 0.05. Student’s two-tailed paired *t*-tests were used for comparisons between matched conditions (*P* = 0.0218 for PD-L1^+^ cells); *n* = 4 biologically independent samples per condition, each derived from primary OPC cultures isolated from a different mouse. Source Data

**Extended Data Fig. 8:**
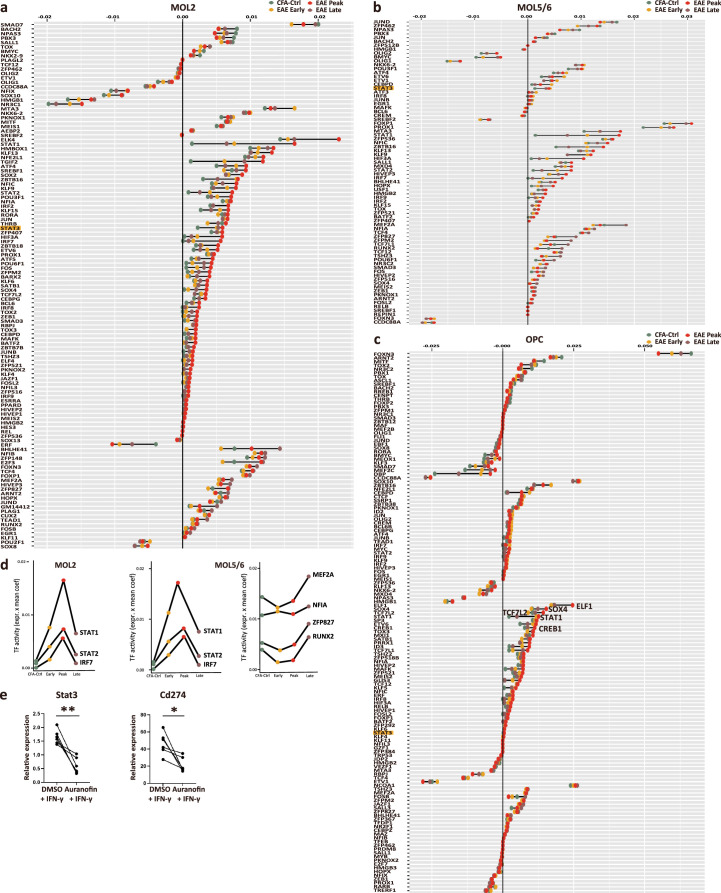
Transcription factor activity combining chromatin accessibility and gene expression in OLGs, and regulation of Cd274 (PD-L1) by STAT3. **a,b,c**, Activity score of selected transcription factor at different stages in MOL2 (**a**), MOL5/6 (**b**), and OPC (**c**). x axis: transcription factor activity score, y axis: transcription factors grouped by time point at which transcription factor activity is the highest. **d**, Activity score of selected transcription factor at different stages in MOL2 and MOL5/6. **e**, Relative expression level of Stat3 and Cd274 in OPCs treated with DMSO or Stat3 inhibitor Auranofin after IFN- γ treatment, measured by qPCR. ** P < 0.01, * P < 0.05; Student’s two-tailed paired t-tests were used for comparisons between matched conditions (P = 0.0031 for Stat3 and P = 0.02 for Cd274); n = 6 independent experiments per condition. Source Data

To obtain insights into the molecular mechanisms mediating the divergent OLG responses in the different stages of EAE, we inferred the global gene regulatory network (GRN)^31^ for OPCs and MOL5/MOL6 and MOL2. We observed changes in the activity of the predicted transcription factors between the different stages of EAE (Fig. 8a–c, Extended Data Fig. 8a–d and Methods). We had previously found that the transcription factor BACH1 negatively regulated the induction of immune-related genes mediated by IFNγ^13^. Interestingly, we observed that its related transcription repressor BACH2 has more activity in CFA-Ctrl mice then in any EAE stage in both MOL2 and MOL5/MOL6 populations (Fig. 8a–c and Extended Data Fig. 8a–d). At early stages of EAE, we found higher activity of NKX6.2 in both MOL2 and MOL5/MOL6, which might be consistent with the transitory regenerative responses observed at early stages. NKX6.2 has an important role in oligodendrogenesis and myelination^32–34^ and has been recently shown to drive OLG specification from induced pluripotent stem cells^35,36^. We also found that STAT3, which has immunosuppression potential^37,38^, showed higher positive activity in MOL5/MOL6 from the early stage, while at the peak stage in MOL2 and OPCs (Fig. 8a,c and Extended Data Fig. 8c)^13^.

We identified 26 transcription factors in MOL5/MOL6 and 23 transcription factors in MOL2, acting mainly with positive activities at the late stage. Among these transcription factors were TCF4, RUNX2, myocyte enhancer factor 2A (MEF2A) and POU homodomain transcription factors (Fig. 8a,b), with functions in nervous system development. In particular within MOL5/MOL6-predicted transcription factors, we found an increased number of candidates related to nervous system development at the late stage, including nuclear factor I A (NFIA) and TCF12, which are involved in the regulation of OL differentiation and maturation^39–41^ (Fig. 8b). Some transcription factors regulating cell differentiation, such as SOX4, were also found to mainly act at the late stage compared to other stages in MOL5/MOL6 (Fig. 8b). However, unlike in MOL5/MOL6, the differentiation-related factors TCF7L2 (ref. ^42^) and SOX4 exhibited the highest activity from the peak stage in OPCs (Extended Data Fig. 8c), suggesting that early CNS regeneration may already be initiated. These results further underscore differential regenerative potential of OLG during the late stages of the disease.

### STAT3 contributes to immunosuppression in MOLs

**Extended Data Fig. 9:**
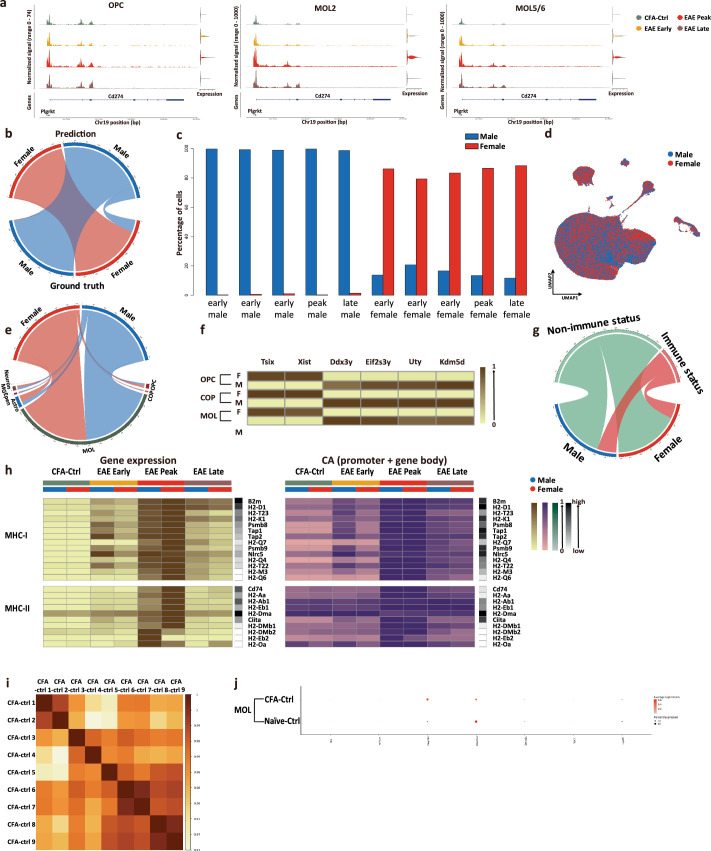
No major differences were found neither in OLG between male and female nor between CFA-Ctrl and naïve-Ctrl groups regarding immune related genes. **a**, Normalized tracks of chromatin accessibility (left) and expression (right) of Cd274 (PD-L1) in OPC, MOL2, and MOL5/6. **b**, Circos plot showing the prediction result (upper semicircle) and downsampled ground truth (bottom semicircle) of the sex. **c**, Bar plot showing the percentage of cells predicted to be male (blue) or female (red) in the samples used to build the sex prediction model. These samples came from different stages of EAE and only contained cells from one gender. **d**, Joint UMAP from the weighted nearest neighbors graph of scRNA-seq and scATAC-seq modalities colored by sex predition. **e**, Circos plot showing the proportion of predicted male and female cells in each cell type, downsampled by sex. **f**, Heatmap showing the expression of differentially expressed genes (log2FC > 1 and pvalue adjusted < 0.01) between male and female in OPC, COP, MOL2, MOL5/6. **g**, Circos plot showing the number of cells with (red) or without (green) immune status for predicted male and female cells (downsampled by sex). **h**, Heatmaps of the expression (left) and chromatin accessibility (right) of MHC-I (upper) and -II (lower) genes in predicted male and female cells at different stages in OLG. The black column on the side represents the gene raw counts. **i**, Correlation matrix showing the Pearson correlation scores of MG between different CFA-Ctrl replicates. **j**, Dot plot showing differentially expressed genes (log2FC > 1 and pvalue adjusted < 0.01) between Naïve-ctrl and CFA-Ctrl in MOL.

STAT3 is a transcription factor that can be involved in immune suppression by, for instance, the upregulation of PD-L1 expression^43,44^. PD-L1 is an immune checkpoint protein whose immune-suppressing effects have been observed across a broad range of cell types^45–55^. We found that both the expression and chromatin accessibility of *Cd274* (which encodes PD-L1) were increased at early and peak stages in OPCs and MOLs (Extended Data Fig. 9a). The increase of PD-L1 expression may thus be stalling the autoimmune response during the early and peak stages of the disease. To investigate whether STAT3 regulates PD-L1 expression in OLG, we knocked down *Stat3* expression using short interfering RNA (siRNA) or inhibited its activity with the inhibitor auranofin^56,57^ in primary cultured neonatal OPCs. We found that both *Stat3* siRNA and auranofin effectively inhibited *Stat3* expression following IFNγ treatment. This inhibition also resulted in reduced *Cd274* expression in response to IFNγ (Fig. 8d and Extended Data Fig. 8e). Furthermore, flow cytometry analysis revealed a decrease in PD-L1 protein expression in OPCs treated with *Stat3* siRNA (Fig. 8e). These findings suggest that certain immune suppression-related genes may be upregulated in OLGs during EAE in response to the inflammatory milieu, attempting to attenuate and regulate the immune response.

## Discussion

The immune plasticity of OLG was first reported about 40 years ago^58^. In addition, MHC class I and MHC class II expression in OLG under inflammatory conditions was further observed in many subsequent studies^59–62^. Recently, with the emergence of single-cell transcriptomics, the concept of imOLG has been further expanded to neuroinflammation in the context of MS, Alzheimer’s disease and aging in several studies^4,5,8,62,63^. We have recently shown that MHC and other immune-associated genes are highly expressed in EAE at the peak stage, with primed chromatin accessibility of some of these genes already in CFA-Ctrl animals^4,13^. In the current study, we applied multiomics scRNA-seq and scATAC-seq to comprehensively profile OLG cellular states throughout EAE, from onset to chronic stages. Our results show that several immune-related genes and their chromatin accessibility levels are already elevated in OLG, in particular in OPCs, at the early stage of EAE, when symptoms start emerging. Furthermore, these loci remain highly accessible even in the late disease stage.

Immune-like OLG emerge early in EAE, before lesions are fully developed^64^. We also observe that the percentage of MHC class II^+^ OLG is similar inside and outside lesions, suggesting that the lesion environment is not essential for the transition of OLG to immune-like states. These findings are consistent with spatiotemporal transcriptomics analysis in EAE, indicating that disease-associated glia can be induced independent of lesions and that the disease-associated MOLs are in close proximity to disease-associated microglia and astrocytes^64^. Thus, it is plausible that the induction of imOLG might be mediated not only by infiltrating immune cells but also by other disease-associated glia or inflammatory environmental factors.

The presence of imOLG at all stages of EAE could suggest differential functions at distinct time points. We observed that only a small percentage of *Sox10*^+^ cells express MHC class II genes. However, even minimal expression can have substantial functional implications. In MS and EAE, OLG are direct targets of the autoimmune response within the CNS. MHC class II expression in OLG might enable them to present antigens and activate CD4⁺ T cells, positioning OLG at the forefront of disease onset and progression. Nevertheless, our results showed immune-repressive pathway activation in MOLs at EAE early and peak stages, aligning with previous findings that OPCs and MOLs express PD-L1 (ref. ^13^). Thus, MOLs could, in principle, activate or, alternatively, block immune responses in the context of EAE. At the early stages, immune MOLs might act by modulating (preventing or initiating/amplifying) the initial neuroinflammatory events underlying the etiology of the disease. At peak stages, given the presence of high numbers of professional antigen-presenting cells, such as microglia and macrophages, the role of immune MOLs might be more subtle and modulatory of the function of these other immune cells. We observe the persistence of MHC class I and MHC class II in OLG in the late stages of EAE, when imOLG might be involved in disease persistence, although a role in reducing inflammatory responses is also possible.

IFNγ treatment of human HeLa cells has been shown to lead to long-term transcriptional memory^65^. Moreover, epigenetic memory of previous inflammatory events has also been observed in mouse epidermal stem cells^66,67^. Disease-associated astrocytes have also been suggested to present epigenetic memory following neuroinflammation^68^, and obesity has also been reported to lead to epigenetic memory in adipocytes, including genes involved in inflammatory signaling^69^. Here, we found that OLG can also retain memory of previous inflammatory insults at a chromatin accessibility level. In the most common course of MS, individuals with relapsing–remitting MS suffer from multiple remissions and relapses over the course of the disease, and the symptoms can worsen after each relapse^1^. Therefore, the observed epigenetic memory of immune-related genes in OPCs may contribute to faster and stronger immune gene expression following the next wave of stimulation during the relapse stage in individuals with MS. This immune epigenetic memory in OLG may contribute to the chronicity of the disease and the difficulty in treating demyelinating diseases.

The heterogeneity of the OL lineage in development has been reported in our previous study^19^, and several subsequent studies have further confirmed the transcriptional and spatial preferences of distinct subpopulations of OL in development and disease^4,7,14,20^. In our analysis, we identified previously reported mature resting subtypes. The percentage of cells with an immune profile was higher in MOL2 than in MOL5/MOL6. The expression of immune-related genes in MOL5/MOL6 decreased considerably at the late stage compared to the early stages. Together, the immune characteristics of MOL2 in the spinal cord of mice with EAE are stronger than those of MOL5/MOL6. MOL5/MOL6 are more enriched in the gray matter of the spinal cord, whereas MOL2 are more preferentially located within the white matter of the spinal cord, where immune infiltrates and lesions are the most prominent^14,70^. The difference in the domain distribution between MOL2 and MOL5/MOL6, in combination with intrinsic factors, may thus contribute to the heterogeneity of MOL in response to the inflammatory environment and higher immune characteristics of MOL2.

We observed notable disparities in CNS development functions between MOL2 and MOL5/MOL6. We saw that some genes related to CNS development always stay in a coupled-on stage in MOL5/MOL6 but not in MOL2, which suggests a more central role of MOL5/MOL6 in myelin maintenance and stability than MOL2 in disease, especially at the chronic stage. However, our results do not provide a definitive answer regarding whether MOL5/MOL6 at the late stages are newly generated or resilient cells that were already present before disease development. One hypothesis is that MOL5/MOL6 are newly generated and possess a stronger ability for CNS development. Alternatively, it is still possible that these MOL5/MOL6 survive in a severe inflammatory environment and contribute to remyelination once the inflammation in the CNS is diminished. To address this question, lineage tracing experiments need to be conducted in future studies.

Our study provides a unique resource for understanding the intricate mechanisms underlying the gene regulation and chromatin accessibility of OLG in the context of MS using an animal model. In particular, our study sheds light on the distinct fates of MOL2 and MOL5/MOL6 during disease evolution, indicating that therapeutic strategies targeting MOLs need to be specific for the different populations. The road to fully understand the complex interplay of transcriptomics and epigenomics in MS requires further exploration, such as spatial resolution, which may provide a more comprehensive and accurate picture of the disease.

### Limitations of the study

Although our multiome analysis gives insights into the epigenetic potential and memory in OLG during the time course of the disease, at the onset of the disease, the inclusion of intermediate time points might allow further granularity and uncovering of additional cellular states in OLG. We also show that OPCs in vitro can acquire epigenetic memory in the form of chromatin accessibility at immune-related genes. Although it is possible that this epigenetic memory occurs in MOLs, it is challenging to assess this in vitro due to cell death of MOLs after prolonged culture in vitro. Future studies might elucidate whether this epigenetic memory also occurs in adult OPCs and MOLs in vivo and whether it plays a role after the second inflammatory challenge, as, for instance, in relapsing–remitting EAE models. The observed priming of OL differentiation and myelination gene programs in MOL5/MOL6, particularly at the early stages of EAE, suggest that there might be a window of opportunity for the promotion of MOL-associated remyelination in MS. Nevertheless, our results indicate only potential given the nature of epigenomic data, and it is thus unclear whether it is possible to harness this MOL capability to promote remyelination. A deeper analysis of the epigenetic landscape at a single-cell level by examining, for instance, activating and repressive histone modifications^71^ or DNA methylation might further elucidate mechanisms that could lead to the activation of these gene programs and promote remyelination in the context of MS.

## Methods

### Animals

The present study followed some applicable aspects of the PREPARE and ARRIVE^72,73^ planning guidelines checklist such as the formulation of the in vivo study, dialog between scientists and the animal facility and quality control of the in vivo components in the study. All animals were born, bred, housed and subjected to experimental treatment at Karolinska Institutet, Comparative Medicine Biomedicum animal facility for research on small rodents (KM-B). *Sox10*:*cre-RCE*:*loxP* (eGFP) transgenic mice were used in this study and were originally obtained by crossing mice with Cre recombinase under the control of the *Sox10* promoter (The Jackson Laboratories, 025807; with a C57BL/6 genetic background) with reporter mice *RCE*:*loxP*-*EGFP* (with a CD1 background, 32037-JAX) to label the complete OL lineage. Breedings were performed with *cre* allele females and non-*cre* carrier males, with one male and up to two females. Breeding males carrying a hemizygous *cre* allele, along with the reporter allele, with non-*cre* females was avoided because it resulted in progeny expressing eGFP in all cells. Mice included in the experiment were heterozygotes and between 9 and 13 weeks old; both males and females were included. None of the experimental animals in this study were subjected to previous procedures. The following housing conditions were used: dawn 06:00–07:00, daylight 07:00–18:00, dusk 18:00–19:00 and night 19:00–06:00. A maximum of five adult mice was housed per individually ventilated cage of type II (IVC seal safe GM500, Tecniplast). All animals were free from mouse viral pathogens, ectoparasites and endoparasites and mouse bacteria pathogens. Animal health surveillance was performed every third month according to the Federation of European Laboratory Animal Science Association recommendations^74^. Three basic and one complete Federation of European Laboratory Animal Science Association tests were performed per year (health monitoring was performed via PCR on exhaust air filter dust and by serology from sentinel animals). General housing parameters, such as relative humidity, temperature and ventilation, followed the European Convention for the Protection of Vertebrate Animals Used for Experimental and Other Scientific Purposes Treaty ETS 123, Strasbourg 18.03.1996/01.01.1991. Briefly, a relative air humidity of 55% ± 10% and temperature of 22 °C were maintain, and air quality was controlled with the use of stand-alone air handling units supplemented with HEPA-filtrated air. Monitoring of husbandry parameters was performed using ScanClime (Scanbur) units. Cages contained hardwood bedding (TAPVEI), nesting material, shredded paper, gnawing sticks and card box shelter (Scanbur). The mice received a regular chow diet (R70 or R34, Lantmännen Lantbruk or CRM(P) SDS or CRM(P), SAFE). Water was provided by using a water bottle, which was changed weekly (water quality was assessed by 1SO 6222, SS EN-ISO 9308-2:2014 and SS EN-ISO 14189:2016 methods, Eurofins). Cages were changed once every week. All cage changes were performed in a laminar air flow cabinet (NinoSafe MCCU mobile cage changing unit) provided with a HEPA H14 EN1822 filter (0.3-μm particle size). Facility personnel wore dedicated scrubs, socks and shoes. Respiratory masks were used when working outside of the laminar air flow cabinet. Animals of both sexes were assigned to different experimental groups by randomization using the GraphPad randomization tool (GraphPad by Dotmatics).

All experimental procedures on animals were performed following the European Directive 2010/63/EU, local Swedish directive L150/SJVFS/2019:9, Saknr L150 and Karolinska Institutet complementary guidelines for procurement and use of laboratory animals (Dnr. 1937/03-640) and Karolinska Institutet Comparative Medicine veterinary guidelines and plans (version 2020/12/18). The procedures described were approved by the local committee for ethical experiments on laboratory animals in Sweden (Stockholms Norra Djurförsöksetiska nämnd), license numbers 1995-2019 and 7029-2020.

### EAE

For EAE induction, animals were injected subcutaneously with an emulsion of MOG_35–55_ in CFA (Hooke Laboratories, EK-2110; containing 1 mg of MOG_35–55_ per ml emulsion and 2–5 mg of killed mycobacterium tuberculosis H37Ra per ml emulsion, Hooke Laboratories), followed by the intraperitoneal injection of pertussis toxin (Hooke Laboratories, included in the induction kits) in 1× PBS (Gibco, 10010023) on days 0 and 1 (200–225 ng per animal, adjusted by lot according to the manufacturer’s instructions). Scores of EAE were graded according to the following criteria: 0, asymptomatic; 1, limp tail or titubation; 2, limp tail and weakness of hindlimbs; 3, limp tail and complete paralysis of hindlimbs; 4, limp tail, complete paralysis of two hindlimbs with forelimb involvement; 5, moribund or dead; 0.5 for intermediate symptoms. Investigators were not blinded to group allocation and were aware of whether animals were assigned to the control or EAE group during both experimentation and outcome assessment. Accordingly, data collection and analysis were not blindly performed to the conditions of the experiments.

CFA-Ctrl mice were injected subcutaneously with control emulsion containing CFA but without MOG_35–55_ (Hooke Laboratories, CK-2110), followed by the administration of pertussis toxin in PBS on days 0 and 1 (200–225 ng per animal, adjusted by lot according to the manufacturer’s instructions). Spinal cords from mice with EAE were collected at the (1) early stage (days 8–9 after injection, score of 0–0.5, 14 mice in 7 multiome experiments), (2) peak stage (days 14–15, score of 3, 10 mice in 5 multiome experiments) or (3) late/chronic stage (days 37–40, score of 2–2.5, 14 mice in 6 multiome experiments). Spinal cord samples from CFA-Ctrl mice were also collected from the same stages (early stage, peak stage and late stage) with a score of 0 (at least four mice in two multiome experiments for each stage), alongside spinal cord tissues from noninduced Naive-Ctrl animals (3-month-old mice from the same strain, six mice in three multiome experiments). Scores for mice with EAE and CFA-Ctrl mice were plotted using GraphPad Prism version 9.0.0.

### Tissue dissociation, FACS and single-cell multiome RNA-seq and ATAC-seq

At the early, peak and late stages, mice were perfused with cold PBS, and spinal cords were collected. Spinal cord tissues were then dissociated into a single-cell suspension according to the manufacturer’s protocol for the Adult Brain Dissociation kit, mouse and rat (Miltenyi Biotec, 130-107-677; we did not perform the red blood cell removal step because the majority of red blood cells had been removed after PBS perfusion). For the debris removal step, most of the samples were processed with 38% Percoll except two Naive-Ctrl samples, two EAE peak-stage samples and two EAE late-stage samples, which were processed with debris removal solution (Miltenyi Biotec, 130-107-677) according to the manufacturer’s protocol, to enrich more OPCs. Spinal cord single eGFP^+^ cells were enriched with a BD FACS Aria III Cell Sorter (BD Biosciences).

The cells were then lysed and washed according to the demonstrated protocol for Nuclei Isolation for Single Cell Multiome ATAC + Gene Expression Sequencing (10x Genomics, CG000365), with the following modifications. Cells were centrifuged for 10 min at 300*g* and 4 °C, resuspended in lysis buffer (containing 10 mM Tris-HCl (pH 7.4), 10 mM NaCl, 3 mM MgCl_2_, 0.01% Tween-20, 0.01% IGEPAL (CA-630), 0.001% digitonin, 1% bovine serum albumin (BSA), 1 mM dithiothreitol (DTT) and 1 U μl^−1^ RNase inhibitor) and incubated on ice for 3 min. After the incubation, wash buffer (containing 10 mM Tris-HCl (pH 7.4), 10 mM NaCl, 3 mM MgCl_2_, 0.1% Tween-20, 1% BSA, 1 mM DTT and 1 U μl^−1^ RNase inhibitor) was added on top without mixing. The nuclei were centrifuged for 5 min at 500*g* and 4 °C. Nuclei were washed once in wash buffer, followed by another wash with diluted 1× Nuclei buffer (10x Genomics, PN-1000283) containing 1% BSA, 1 mM DTT and 1 U μl^−1^ RNase inhibitor. Chromium Next GEM Single Cell Multiome ATAC + Gene Expression chemistry (10x Genomics, PN-1000283) was then applied to yield single-cell ATAC and RNA libraries. Twenty-six mice with EAE (8 early-stage mice, 8 peak-stage mice and 10 late-stage mice), 12 CFA-Ctrl mice (4 early-stage mice, 4 peak-stage mice and 4 late-stage mice) and 4 Naive-Ctrl mice were used for independent replicates. Libraries were sequenced on an Illumina Novaseq 6000 with a 50–8–24–49 read setup for ATAC (minimum of 25,000 read pairs per cell) and a 28–10–10–90 read setup for RNA (minimum of 20,000 read pairs per cell).

Both male and female mice were used in our study. Most of the samples contained cells from one male mouse and one female mouse, except two early-stage EAE samples (one only contained male mice, another only contained female mice), two peak-stage EAE samples (one only contained male mice, another only contained female mice) and two late-stage EAE samples (one only contained male mice, another only contained female mice) for establishing a sex prediction model and validation.

### RNAscope, immunohistochemistry and confocal microscopy

RNAscope ISH was performed on 14-μm spinal cord sections from the lumbar spinal cord of control mice and mice with EAE (*n* = 3 for each condition) with probes for mouse *Sox10* (ACD, 435931) and *H2-Ab1* (ACD, 414731-C2). The RNAscope ISH protocol for sections was performed following the manufacturer’s instructions with minor modifications (RNAscope Multiplex Fluorescent Detection Reagents v2, 323110). Tissue sections were incubated in 1× target retrieval reagent (RNAscope Target Retrieval Reagents, 322000) for 5 min at 98 °C, followed two 2-min washes with DNase/RNase-free water. The samples were then incubated with protease IV (RNAscope Protease III and Protease IV Reagents, 322340) at room temperature for 20 min, followed by two 2-min washes with DNase/RNase-free water. The sections were hybridized with probes (C1:C2/C3 in a 1:50 dilution) for 2 h at 40 °C and washed twice with wash buffer (RNAscope Wash Buffer Reagents, 310091). Amplification steps were performed by incubating with v2Amp1 (30 min), v2Amp2 (30 min) and v2Amp3 (15 min) (RNAscope Multiplex Fluorescent Detection Reagents v2, 323110) at 40 °C, with two 2-min washes with wash buffer in between steps. The sections were incubated with v2-HRP-C1 for 15 min at 40 °C, and after two 2-min washes with wash buffer, tyramide signal amplification (TSA)-conjugated fluorophores (1:1,500 dilution in TSA buffer; RNAscope Multiplex TSA Buffer, 322810) were added and incubated for 30 min at 40 °C. The sections were washed twice with wash buffer and incubated with HRP blocker for 30 min at 40 °C. The v2-HRP, TSA-conjugated fluorophore and HRP blocker steps were then repeated for C2 and C3 channels, if applicable. At the end, the sections were incubated with DAPI (Sigma, D9542; 1:5,000) for 5 min and washed with PBS with 0.05% Tween-20 (VWR, 9005-64-5) for 2 min.

For immunohistochemistry, after blocking with 5% normal donkey serum (Sigma) in 0.3% PBS/Triton X-100 for 1 h at room temperature, spinal cord sections were incubated overnight at 4 °C in the following primary antibodies: anti-MHC class II (Invitrogen, 14-5321-85, rat 1:50, clone M5/114.15.2) and anti-GFP (Abcam, ab13970, chicken 1:200) diluted in PBS/0.05% Tween-20/2% normal donkey serum. After washing the sections with PBS/0.05% Tween-20, secondary Alexa Fluor-conjugated antibodies (goat anti-rat (for MHC class II, 1:1,000; Invitrogen, A21434) and goat anti-chicken (for GFP, 1:1,000; Abcam, ab150169)) diluted in PBS/0.05% Tween-20/2% normal donkey serum were incubated for 2 h at room temperature. Slides were counterstained with DAPI, mounted with mounting medium and maintained at 4 °C until further microscopic analysis.

Images were acquired using a Zeiss LSM800 confocal microscope (RNAscope) and Zeiss LSM980 microscope (immunohistochemistry) and processed in Fiji/ImageJ. For RNAscope, six ×20 randomly selected fields per mouse (three from lesions and three from nonlesions) were chosen for quantification. The percentage of MHC class II^+^ OLG was significantly higher at the peak stage (lesion: 5.33 ± 0.23%, nonlesion: 5.32 ± 0.23%) than at both early stage (lesion: 0.49 ± 0.849; nonlesion: 0.384 ± 0.333%) and late stage (lesion: 2.54 ± 0.23; nonlesion: 2.71 ± 0.32%) in both lesion (*P* = 0.0001) and nonlesion (*P* = 0.0001) areas (Fig. 2j). A two-way ANOVA with a Tukey’s multiple comparisons test was performed using GraphPad Prism version 9.0.0 for comparing the percentages of *Sox10*^+^*H2-Ab1*^+^ cells between different time points and between lesion and nonlesion.

### Primary OPC culture

*Sox10*:*cre-RCE*:*loxP* (eGFP) transgenic mice were used. The brains of postnatal days 3–6 pups were dissociated using a Neural Tissue Dissociation kit (Miltenyi Biotec, 130-092-628), according to the manufacturer’s protocol. OPCs were obtained with MACS with CD140a microbeads following the manufacturer’s protocol (CD140a Microbead kit, Miltenyi Biotec, 130-101-547). Cells were seeded in poly-L-lysine-coated (Sigma, P4707) dishes and grown on OPC proliferation medium comprising DMEM/F-12/GlutaMAX (Thermo Fisher, 10565018), N2 medium (Thermo Fisher, 17502048), penicillin–streptomycin (Thermo Fisher, 15140122), NeuroBrew (Miltenyi, 130-097-263), bFGF (40 ng ml^−1^; R&D, 233-FB) and PDGF-AA (20 ng ml^−1^; Peprotech, 315-17).

### Oli-neu cell culture

Oli-neu cells (obtained from J. Trotter, Johannes Gutenberg University) were seeded in poly-L-lysine-coated (Sigma, P4707) dishes and grown on proliferation medium comprising DMEM (Gibco, 41966029) supplemented with 1× N2 supplement (Thermo Fisher, 17502048), 1× penicillin–streptomycin–glutamine (Gibco, 10378016), 340 ng ml^−1^ T3 (Sigma, T6397), 400 ng ml^−1^ T4 (Sigma, 89430), 10 ng ml^−1^ bFGF (R&D, 233-FB), 1 ng ml^−1^ PDGF-BB (R&D, 520-BB) and 0.5% fetal bovine serum (Gibco, 10500064).

### IFNγ treatment, RT–qPCR, bulk RNA-seq and bulk ATAC-seq

For the 2× IFNγ treatment, primary OPCs (*n* = 2) and Oli-neu cells (*n* = 3) were initially treated with IFNγ (100 ng ml^−1^; R&D, 485-MI-100) for 24 h. Afterward, the cells were cultured in proliferation medium without IFNγ for 96 h, and then a second dose of IFNγ was administered for an additional 24 h. For the 1× IFNγ treatment, cells were treated with IFNγ only at the time when the 2× IFNγ-treated cells received their second dose. RNA extraction was performed using an RNeasy kit (Qiagen, 74106), and RNA-seq libraries were prepared using Stranded Total RNA Prep, Ligation with Ribo-Zero Plus (Illumina, 20040525), according to the manufacturer’s protocol. Libraries were sequenced on an Illumina NovaSeq X, with a 75–10–10–75 read setup.

RNA extraction was performed using an RNeasy kit (Qiagen, 74106), followed by cDNA synthesis using a High-Capacity cDNA Reverse Transcription kit (Thermo Fisher, 4368814), according to the manufacturers’ protocols. RT–qPCR was performed on a QuantStudio 5 using SYBR Green Master Mix (Thermo Fisher, 4385617; Supplementary Table 10). Relative gene expression was calculated using the change in cycling threshold (${2}^{-\varDelta \varDelta \,{C}_{{\rm{t}}}}$) method, normalizing to the housekeeping gene *Gapdh*.

ATAC-seq was performed as previously described^11^, with minor adaptations. For each condition, 50,000 primary OPCs were collected, washed with PBS and lysed with 50 μl of lysis buffer (10 mM Tris-HCl (pH 7.4), 10 mM NaCl, 3 mM MgCl_2_, 0.1% IGEPAL (CA-630), 0.1% Tween-20 and 0.01% digitonin) on ice for 3 min. After lysis, 1 ml of wash buffer (10 mM Tris-HCl (pH 7.4), 10 mM NaCl, 3 mM MgCl_2_ and 0.1% Tween-20) was added, and the nuclei were pelleted by centrifugation at 500*g* for 10 min at 4 °C. After aspirating the supernatant, the nuclei were resuspended in 50 μl of transposition mix (25 μl of 2× TD buffer (Tagment DNA Buffer), 16.5 μl of PBS, 0.5 μl of 1% digitonin, 0.5 μl of 10% Tween-20, 2.5 μl of Tn5 Transposase (final 100 nM) and 5 μl of nuclease-free water) and incubated for 30 min at 37 °C in a thermomixer with mixing at 1,000 rpm. The DNA was purified both before and after PCR using a Zymo DNA Clean and Concentrator-5 kit (Zymo Research). Libraries were sequenced on an Illumina NovaSeq X with a 51–8–8–50 read setup.

### Transcription factor knockdown/inhibition, RT–qPCR and flow cytometry

For transcription factor knockdown, siGENOME SMARTpool nontargeting control siRNA (D-001206-13-05, Dharmacon) or siRNA targeting *Stat3* (M-040794-00-0005, Dharmacon) was used, which contains a pool of four siRNAs. One milligram of siRNA was diluted in OPTIMEM (31985062, Gibco), mixed with Lipofectamine 2000 (11668027, Invitrogen) and allowed to form complexes for 20 min at room temperature. Primary OPCs were then incubated with the siRNA complexes in OPTIMEM with N2 medium, NeuroBre, bFGF (40 ng ml^−1^) and PDGF-AA (20 ng ml^&minu;1^). After 72 h, IFNγ (100 ng ml^−1^) was added for 24 h. For the STAT3 inhibitor auranofin (Enzo Life Sciences), primary OPCs were cultured in OPC proliferation medium with 2 μM auranofin for 24 h, followed with IFNγ (100 ng ml^−1^) treatment for 24 h.

Specific gene sequences were used for qPCR, and the primer sequences used are as shown in Supplementary Table 10. Relative gene expression was calculated using the ${2}^{-\varDelta \varDelta \,{C}_{{\rm{t}}}}$ method, normalizing to the housekeeping gene *Gapdh*. A two-tailed paired *t*-test was performed using GraphPad Prism version 9.0.0 for comparing the relative expression levels.

For flow cytometry, OPCs were collected and washed with staining buffer (PBS/0.5% BSA). The cells were then stained using a LIVE/DEAD Fixable Near-IR Dead Cell Stain kit (for 633- or 635-nm excitation, Invitrogen) to determine cell viability, followed by staining of PD-L1-APC-conjugated antibody (1:100; BioLegend, 124312, clone 10F.9G2) for 30 min at 4 °C and washing once with staining buffer. Cellular fluorescence was measured with CantoII (BD Biosciences). Forward- and side-scatter parameters were used for the exclusion of doublets. Data were analyzed with FlowJo software 10.8.1 (TreeStar).

### Raw data processing

In total, seven batches were collected from the sequencing facility. Fastq files from 28 samples were processed throughout the 10x Genomics standard pipeline. Gene expression and chromatin accessibility libraries were inputted into cellranger-arc2 (ref. ^75^) ‘count’ v2.0.2 with default settings to align the biological readouts on the associated mm10 reference genome v2020-A-2.0.0. Sample aggregation of both transcriptomic and genomics metrics was done using the ‘aggr’ of the same CellRanger executable file, without normalization.

The aggregated count matrix and fragments file were loaded into R v4.3.2 (https://www.R-project.org). The former was lodged into a Seurat v5.0.3 (ref. ^76^) assay, whereas the latter was accommodated into a Signac v1.13.0 (ref. ^77^) chromatin assay associated with Ensembl^78^ base annotation v79_2.99.0 for mice where University of California, Santa Cruz, nomenclature was applied to provide gene name readability.

If not specifically mentioned, the following pipeline descriptions belong to either the Seurat or Signac package.

### Removal of ambient RNA

Cell-free mRNA contamination was removed from each droplet using Cellbender v0.3.0 (ref. ^79^). Genes called in all samples by Cellbender were included into the feature list. Potential cells called in both CellRanger and Cellbender were included in the cell list. In total, 181,674 cells were retrieved. Most of the genes affected by the ambient RNA removal were canonical genes and ribosomal genes (120 genes with a log_2_ (FC) greater than 1).

### Filtering of low-quality cells

After determining that each cell could be uniquely identified, quality metrics for both gene expression and peak accessibility were calculated. Among others, the mitochondrial ratio, cell cycle score, nucleosome signal and TSS enrichment were generated to support the quality control cutoffs.

Minimum and maximum outliers in counts, detected genes, TSS enrichment and percentage of mitochondrial information were removed from the analysis (Supplementary Methods).

### Peak calling

The aggregated fragments file was used in MACS2 v3.0.0 (ref. ^80^) to proceed to a peak call. Peaks falling in nonstandard chromosomes or overlapping genomic blacklist regions from the mm10 genome were discarded. The fraction of reads in peaks was then calculated for each cell, and the mean value for each sample ranged from 0.64 to 0.79.

### Peak annotation

The annotation of each peak was performed using the Ensembl base annotation v79_2.99.0 for mice, following the 10x Genomics guideline (https://www.10xgenomics.com/support/software/cell-ranger-arc/latest/analysis/peak-annotations), and saved in the same format as cellranger-arc output. In total, 976,949 entries were registered, with 20,611 promoter, 939,715 distal (10% inside the gene body) and 16,623 intergenic hits.

### Peak connection

To consider a peak associated with a given gene, the function LinkPeaks from Signac was used with a *P* value cutoff at 0.05. The *P* values were corrected using the Benjamini–Hochberg method, and only associations with a *P* value adjusted lower than 0.01 were selected for downstream analysis. Peak coaccessibility was performed using Cicero v1.3.9, and *P* values were calculated on *z* scores of coaccessibility scores, adjusted using the Benjamini–Hochberg method and selected if less than 0.01 (Supplementary Methods).

### Gene activity

The computation of fragment counts per cell in the gene body and promoter region (gene activity) was performed with the GeneActivity function from Signac, fetching all biotypes and extending 500 bp upstream of the TSS to catch the promoter region. All other parameters were set to default. This matrix was used later to infer chromatin accessibility for each gene.

### Doublet detection

The detection of putative events where more than one cell in the same droplet occurs was performed using DoubletFinder v2.0.4 (ref. ^81^).

Most of the samples drew less than 1% of doublets, but eight samples drew 1.49, 1.59, 1.88, 2.35, 3.33, 5.08, 7.85 and 20.86% of doublets. Using the multiplet rate provided by 10x Genomics (https://kb.10xgenomics.com/hc/en-us/articles/360001378811-What-is-the-maximum-number-of-cells-that-can-be-profiled), we were able to measure a 0.8%/8% theoretical number of doublets. Predicted doublets were removed from the downstream analysis (Supplementary Methods).

### Sex classification model

In total, 69,152 cells were sequenced with a priori sex annotation. This knowledge was used to create a machine-learning sex classifier to investigate sex-dependent transcriptomic or epigenetic differences. Equal numbers of cells for each sex (30,940 cells) were extracted from the main object to a sex object to build the model. Mouse genes and positions from chromosomes X and Y were extracted from Ensembl v79_2.99.0 (ref. ^78^).

The validation on the testing dataset yielded an accuracy of 95.4%, a sensitivity of 91.0% and a specificity of 99.7%. An insignificant number of male cells was annotated as female cells, while a few female cells were assigned as male cells. We then used the newly created model to process each cell of the main object. Correction of misassigned cells (8.32%) from a priori sex-annotated samples was performed (Supplementary Methods).

Some studies have reported that immune responses are different in many aspects between males and females in both MS and its animal models^82,83^. Because most of our samples were mixed with one male mouse and one female mouse, we created a sex prediction model based on the expression of sex-related genes (Methods). This sex prediction model was validated with samples containing cells from only male or female mice with EAE, with an accuracy of 95.3% on the validation dataset. There was better prediction accuracy in the male sample (99.48%) than in the female sample (86.13%; Extended Data Fig. 9b,c). We applied this sex prediction model to the entire dataset, annotated the sex of the cells (Extended Data Fig. 9d,e) and compared the differences between male and female OLG based on the sex prediction results. For OLG subpopulations, apart from sex-related genes, such as X inactive specific transcript (*Xist* and *Tsix*), ubiquitously transcribed tetratricopeptide repeat containing Y-linked (*Uty*), eukaryotic translation initiation factor 2 subunit 3 structural gene Y-linked (*Eif2s3y*), dead-box helicase 3 Y-linked (*Ddx3y*) and lysine demethylase 5D (*Kdm5d*), no other genes were found to be differentially expressed between males and females (Extended Data Fig. 9f). Accordingly, we found no major differences between the OLG of males and females at the gene expression level in our data. We also did not observe a significant difference in the percentages of imOLG between male and female mice (Extended Data Fig. 9f–h). Thus, our data indicate that OLG responses to the neuroinflammatory environment in EAE are not characterized by sexual dimorphism.

### Normalization

Each feature of each cell of the gene count matrix was divided by the total counts of that cell and multiplied by a scale factor of 10,000. The obtained scaled matrix was then natural-log-transformed. The peak count matrix was processed through a term frequency inverse document frequency (TF-IDF) normalization, which computed a log (TF × IDF) with a scale factor of 10,000. Each feature of each cell of the gene activity matrix was divided by the total counts of that cell and multiplied by a scale factor of 10,000. The obtained scaled matrix was then natural-log transformed.

### Significant features selection

To proceed to the reduction of the dataset dimensions, the 2,000 most variable genes were selected via variance stabilizing transformation, and 95% of the most common peaks were also selected. The expression of each gene was transformed by subtracting its average expression and scaled by dividing its standard deviation.

### Dimension reduction

Reduction of the dimensionality of the expression matrix on the 2,000 most variable genes was done by running a principal component (PC) analysis. The first 30 PCs, representing most of the diversity of the dataset, were selected by the elbow plot method. The partial singular value decomposition was used to reduce the dimensions of the most common peaks, and 12 of the first latent semantic indexing (LSI) layers were selected via the elbow method. However, the first LSI was removed from the selected dimensions due to its high correlation with chromatin capture depth, which would bias the investigation of the chromatin opening binarity.

### Construction of the nearest neighbor graph

For both PCs and LSI, the graphs were made with the ‘annoy’ method with Euclidean distances and 50 trees. A ‘*k*’ for the *k*-nearest neighbor algorithm was set to 20. For the construction of the graph based on PCs, an acceptable Jaccard index was set to 0. For the multimodal nearest neighbor graph, the same numbers of PCs and LSIs were used. A ‘*k*’ for the *k*-nearest neighbor algorithm was set to 20, 200 approximate neighbors were computed, and the cutoff to discard the edge in the shared nearest neighbor graph was set to 0.

### Cluster determination

Unsupervised clustering of the nearest neighbor graphs was performed using the Louvain algorithm. The resolution of each clustering was assessed using the ‘clustree’ R package iterating over different resolutions to pinpoint the most stable clustering resolution. Resolutions of 1, 0.4 and 1.2 were selected, generating 19, 13 and 24 clusters, for gene expression, peak accessibility and joined modality, respectively. Of note, an undetermined cluster specific at 97.86% for a unique sample at the early time point was standing out of all other clusters. All 1,973 cells from this cluster were therefore removed from the downstream analysis.

### Cell projection

In all visualizations of the cells, despite the high dimensionality of the data, the nearest neighbor graphs were reduced to only two dimensions using UMAP with the appropriate number of PCs and LSIs (‘Dimension reduction’), and other values were set as default.

### Batch effect correction

As sample libraries were not prepared on the same day and sequenced in the same sequencing run, and technical artifacts might arise and generate variability not related to biology in the dataset. However, accounting for such disequilibrium, without removing the subtle differences between time points, was challenging. Batch effect correction methods such as Harmony^84^ or Scanorama^85^ with soft parameters completely masked the differences between time points. Moreover, sample integration with ‘cca’ and ‘rpca’ was also overcorrecting time-point-specific cell populations. Although the popular batch effect methods were not suitable for our particular dataset, we relied on the strength that every sequencing run possessed at least one CFA-Ctrl sample (except on late time point and Naive-Ctrl samples). Thus, we investigated if the cells in each CFA-Ctrl cluster were well mixed across the different replicates. All 14 major CFA-Ctrl clusters contained cells from all replicates. To analyze further at the cell mixing level, a local inverse Simpson’s index (LISI) from Harmony v1.2.0, accounting for categorical variable diversity, was calculated for an equivalent number of cells in each CFA-Ctrl replicate, using the first two components of the joint projected UMAP. This score, initially ranging from 1 to the number of replicates, was normalized to a scale of 0 to 1. For CFA-Ctrl, 63% of the cells maintained a LISI of at least 0.5. The same strategy was applied to the Naive-Ctrl samples, where 15 of the 17 major Naive-Ctrl clusters mainly contained cells ascending from the two replicates; one homogeneous cluster was coming from a microglia cluster and the other one was from a pericyte population. At the single-cell level, 76% of the cells maintained a LISI of at least 0.5. For all EAE time points, the same method was applied, yet the calculated LISI was lower than CFA-Ctrl or Naive-Ctrl (EAE score equal to 0), probably due to the heterogeneity of the EAE score at the sample collection within each time point (46% for early, 51% for peak and 49% for late). Although this method does not allow us to mitigate potential hidden batch effects, it provides a well-balanced cell distribution at a consistent EAE score.

### Label transfer

After removing cells with less than 200 detected genes, more than 8,000 detected genes and less than 10% of reads matching the mitochondrial genome using a dataset from the literature^4^, the transcriptomes of the remaining cells were normalized and scaled using SCTransform from Seurat. A label transfer was then performed from the literature annotation to the present dataset based on the transcriptional profile of selected anchors via the FindTransferAnchors function using canonical correlation analysis, the ten first dimensions of the PC analysis and recomputing of the residual with the reference SCT model parameters. The label prediction was run using TransferData with the previously determined anchors set and the canonical correlation analysis reduction to weight the anchors. For each cell, the highest prediction score was selected to make the annotation.

### Cell annotation

Cell-type annotation was assessed per cluster on the joined graph clustering resolution, and label transfer outputs, cell-type marker gene expression and chromatin accessibility were taken into consideration. More refined cell types for the MOL population were later considered.

### Immune score

Exhaustive lists of immune-related genes published in Meijer et al.^13^, specific to ‘immune response’ (GO:0002250) and ‘immune system process’ (GO:0002376), were used to evaluate the immune capability of each cell. Cells from Naive-Ctrl animals were used as the background signal. After removing unexpressed genes in our dataset, subcategories with genes related to positive regulation of immune response, negative regulation of immune response, antigen processing and presentation, positive regulation of type I/II IFN-mediated signaling pathway, positive regulation of cytokine production, complement activation, CD4^+^ T cell-related immune response and CD8^+^ T cell-related immune response were extracted from the immune gene list, respectively, using the MGI immune database (https://www.informatics.jax.org/vocab/gene_ontology).

For each cell type, immune scores were calculated for each immune subcategory and as a whole, using the AddModuleScore function from Seurat with default parameters. All scores were scaled from 0 to 1, and the probability of each cell following the immune score distribution of the Naive-Ctrl population was processed using the following distribution function, where *μ* and *σ* are the mean and standard deviation of the immune score distribution of the Naive-Ctrl population, respectively:

$$\Pr \left(X\le x\right)=F\left(x\right)=\frac{1}{2}\left[1+{erf}\left(\frac{x-\mu }{\sigma \sqrt{2}}\right)\right]$$

Under the null hypothesis that each cell follows the immune score distribution of Naive-Ctrl cells in a given population, each cell with a *P* value less than 0.05, rejecting the null hypothesis, was classified as ‘immune’. Cells accepting the alternative hypothesis (*P* value greater than 0.05), sharing a relationship between the immune score of a given cell and the immune score distribution of the Naive-Ctrl population, were classified as ‘nonimmune’. Due to the lack of cells annotated as neurons in Naive-Ctrl samples, this population was not annotated. The same approach was used on the gene activity matrix to compare transcriptional and epigenetic immune states. An investigation of cell status was also performed using damage-associated gene lists^8^ (95 genes) following the same strategy (Supplementary Table 9).

### Naive-Ctrl versus CFA-Ctrl comparison

To acknowledge the impact of CFA and pertussis toxin in CFA-Ctrl compared to Naive-Ctrl animals, we first performed a Pearson correlation on the gene expression matrix in each cell type between replicates of each time point. We found a high similarity of expression between OLs from CFA-Ctrl time points (Pearson correlation score between CFA-Ctrl replicates: 0.97–0.99; Extended Data Fig. 9i) and Naive-Ctrl replicates (Pearson correlation score between Naive-Ctrl replicates: 0.96–0.99). For OPCs, microglia and astrocytes, the correlation scores were slightly lower (OPCs, CFA-Ctrl replicates: 0.45–0.96, Naive-Ctrl replicates 0.85–0.98; microglia, CFA-Ctrl replicates: 0.69–0.92, Naive-Ctrl replicates: 0.80–0.92; astrocytes, CFA-Ctrl replicates: 0.84–0.99, Naive-Ctrl replicates 0.85–0.95). We did not compare the difference between CFA-Ctrl replicates in neurons, ependymal cells and pericytes due to low cell numbers. Because the cells we used for sequencing were sorted based on GFP signal, these microglia are likely to be activated microglia that engulf OLs and are therefore more sensitive to ex vivo alteration. The finding that microglia are more sensitive than other CNS cell types has also been confirmed before in another study^86^. We did not compare the difference between CFA-Ctrl replicates in neurons, ependymal cells and pericytes due to low cell numbers.

A gene expression matrix with CFA-Ctrl and Naive-Ctrl replicates was loaded into a SingleCellExperiment v1.24.0 (ref. ^87^) object where genes with less than ten counts were removed. Feature counts were then aggregated across cell types and broken down by sample. Each cell type was processed independently, and samples formed by less than 5 cells and time points formed by less than 30 cells were not considered for downstream investigation. The resulting matrix and associated metadata were loaded into an R object using DESeq2 v1.42.1 (ref. ^88^), with a design formula including sample names in addition to the debris removal method as a covariate. After size factors and dispersion estimations, results tables were extracted per contrast of Naive-Ctrl on CFA-Ctrl. Genes presenting an absolute log_2_ (FC) over 1 with an adjusted *P* value less than 0.01 and a baseMean (the average of the normalized count values, dividing by size factors, taken over all samples) greater than 1 were selected as differentially expressed. Seven genes passed the filters in MOLs (*Adamts1*, *Gli2*, *Lypd6*, *Map7d2*, *Npr1*, *Rnf125* and *Sema3c*; Extended Data Fig. 9j), and one gene was differentially expressed in microglia between CFA-Ctrl and Naive-Ctrl samples (*Csmd3*).

### OL lineage investigation

All cells belonging to the OL lineage were subsetted for fine-tuning annotation. One EAE early time point sample collected on day 8 after immunization with a score of 0 (without any symptom but with weight loss during the disease course) was removed from the analysis due to no EAE symptoms and similar gene expression as CFA-Ctrl. As no major gene expression differences were found between Naive-Ctrl and CFA-Ctrl in the OL lineage population, Naive-Ctrl samples were removed from the downstream analysis. The processing of this OL lineage subset of 120,183 cells was similar to the processing of all cell types. Nevertheless, a few differences must be mentioned, specifically, the first 20 PCs and the first 9 LSIs were selected for graph construction. Additionally, cluster resolutions of 2.2, 0.8 and 3.6 were selected for gene expression, peak accessibility and joined modality, respectively. An in-depth label transfer was performed on the subset with the same methods as previously described using only the OL lineage cells from the literature dataset^4^ (Extended Data Fig. 3c,d). From the 47 potential OL lineage cell clusters on joined graph clustering resolution, we aggregated them using the average hierarchical clustering methods (Supplementary Methods). The OPC cluster was subdivided into three subclusters as we observed two time point-specific groups of OPCs and a third group expressing cycling genes. Each aggregated cluster was manually assigned to the main OL lineage cell types (OPC, COP, MOL1, MOL2 and MOL5/MOL6) using specific gene markers and label transfers. OL lineage cell type subclustering (α, β, γ, δ, ε, ζ, η, θ, ι and κ) was assessed and ordered along their average immune score for each main OL lineage cell type (Extended Data Fig. 4a). For instance, compared to other MOL2 populations, MOL2-ε, predominantly derived from the peak stage of EAE (Fig. 3c), exhibited an enrichment of immune-related genes, including IFN-induced protein with tetratricopeptide repeats 3 (*Ifit3*), *Nlrc5* and IFNγ-induced GTPase (*Igtp*), among others (Supplementary Table 2). MOL2-α, which mainly came from CFA-Ctrl and EAE at the early stage, expressed higher lipid metabolic process genes, such as 3-hydroxy-3-methylglutaryl-CoA synthase 1 (*Hmgcs1*), cytochrome P450 family 27 subfamily A member 1 (*Cyp27a1*) and squalene epoxidase (*Sqle*; Supplementary Table 2). Oligodendrocytic myelin paranodal and inner loop protein (*Opalin*), which is associated with OL differentiation, was enriched in MOL5/MOL6-β. A major increase in the expression of actin cytoskeleton organization-related genes, such as actin binding LIM protein family member 2 (*Ablim2*), NCK-associated protein 5 (*Nckap5*) and prickle planar cell polarity protein 1 (*Prickle1*), was observed in MOL5/MOL6-ζ compared to other MOL5/MOL6 populations (Supplementary Table 2). The majority of MOL2-β and MOL2-γ were derived from late-stage EAE (Fig. 3b,c), with gene markers associated with metabolism and differentiation, like ectonucleotide pyrophosphatase/phosphodiesterase 6 (*Enpp6*) and S100 calcium binding protein B (*S100b*; Supplementary Table 2). In addition, most MOL5/MOL6-α, MOL5/MOL6-β and MOL5/MOL6-γ cells were mainly composed of cells from early-stage CFA-Ctrl and EAE samples, MOL5/MOL6-θ were mainly composed of cells from peak-stage EAE and cells from MOL5/MOL6-ζ and MOL5/MOL6-ι were drawn by late-stage EAE (Fig. 3b,c).

### Multimodality features selection

Transcriptomic and epigenomic variations in specific cell types throughout the disease time course were assessed in a pseudobulk manner. This technique allows us to overcome the sparsity of the datasets and highlight dynamic features within three modalities, gene expression, gene/promoter accessibility and peak accessibility. These tri-omics modalities meet the prerequisites for analyses based on the negative binomial distribution (lack of intrasample variability, most of the features remain stable throughout the tested conditions, raw integer counts to specific genomic locations, sufficient number of replicates). Each modality was loaded into a SingleCellExperiment v1.24.0 (ref. ^87^) object where features with less than ten counts were removed. Feature counts were then aggregated across cell types and broken down by sample. Each cell type was processed independently, and samples formed by less than 5 cells and time points formed by less than 30 cells were not considered for the downstream investigation. The resulting matrix and associated metadata were loaded into an R object using DESeq2 v1.42.1 (ref. ^88^), with a design formula including sample names in addition to the debris removal method as a covariate. After size factors and dispersion estimations, a Wald test was performed followed by a multiple testing correction using the Benjamini–Hochberg method. The results tables were then extracted per contrast. We decided to investigate four contrasts (early/control, peak/early, late/early and peak/late), depicting a broad overview of the disease time course. Features presenting an absolute log_2 _(FC) greater than 1 with an adjusted *P* value of less than 0.01 and a baseMean (the average of the normalized count values, dividing by size factors, taken over all samples) of greater than 1 were selected to be displayed on the heat maps and GO analyses for the modalities represented by gene and promoter and were included into the transcription factor analysis for the modality represented by peaks. Differential gene expression analysis between males and females within each time point was performed similarly, merging gene counts by both sample and sex.

### Multimodality pseudotime

Expression dynamics were assessed using velocyto v0.17.17 (ref. ^29^) to retrieve the number of spliced and unspliced reads in each sample. In addition to the CellRanger output directory, the GTF annotation file used by CellRanger was given, along with a GTF annotation file for mm10 repetitive elements from RepeatMasker (https://www.repeatmasker.org). Resulting loom files were loaded into scVelo^89^ in Python and directly imported via MultiVelo v0.1.3. After merging the samples, OL lineage cells were selected to undergo a log-transformed normalization on the 1,000 most variable features with at least ten read counts.

The chromatin accessibility matrix from Macs2 output was loaded, and peaks were aggregated around each gene using peak annotation (‘Peak annotation’) and feature linkage prediction (‘Peak annotation’) inputted in the aggregate_peaks_10x function. OL lineage cells were selected to undergo a TF-IDF normalization.

The 120,183 cells as well as the 798 genes with both expression and chromatin accessibility signals were picked to compute moments for velocity estimation for each cell across its 30 nearest neighbors calculated from Euclidean distances in the first 30 PC spaces of the expression matrix. Weighted nearest neighbor properties calculated previously on the OL lineage cells were used to smooth the epigenetic modality and were incorporated into RNA velocity to recover chromatin dynamics and carry out enhanced lineage predictions. This last step was done on MOL5/MOL6 and MOL2 populations separately. For each cell type, a new UMAP was generated; velocity, latent time and terminal states were processed; and CFA-Ctrl cells were set as root cells.

MultiVelo classifies, if possible, each gene into two modules of biological dynamics. This tool is anchored in two models for the correlation of gene expression and chromatin accessibility changes within the latent time line: a first model (M1) where chromatin starts closing before the end of transcription and a second model (M2) where chromatin starts closing after the end of transcription. Moreover, the coupled kinetics of the transcriptomic and epigenomic profiles can be used as leverage to predict a current cell state for a given gene. The priming state is considered when the chromatin is opening but no unspliced transcript has yet been detected (brown, chromatin is opening but transcription is not initiated). The couple-on state is selected when the chromatin is open and the number of unspliced transcripts is increasing (pink, chromatin is open and transcription is initiated). The decoupling state is picked when there is a decorrelation between chromatin and unspliced transcripts dynamics. For the first model, the chromatin closes before the end of transcription, whereas for the second model, the number of unspliced transcripts starts decreasing but the chromatin is still open (dark blue, M1: chromatin accessibility starts closing before the end of transcription, M2: chromatin is open but transcription repression begins). The couple-off state is set when the chromatin is closed while the number of unspliced transcripts is collapsing (light blue, chromatin is closed and the number of unspliced reads is dropping; Fig. 7a and Extended Data Fig. 7a,b).

Due to the limited number of OPCs, some genes of interest did not present a complete transcriptional trajectory using MultiVelo. Therefore, we focused on key metrics to highlight their partial kinetics, including unspliced RNA, spliced RNA and chromatin accessibility levels of the same group of immune-related genes as in MOLs. We observed that spliced RNA levels of these immune-related genes were elevated in OPCs both at peak and in a subset of early-stage cells and were accessible at all time points. By contrast, unspliced RNA levels of these genes remained low across time points (Extended Data Fig. 7c,d). Thus, based on OPC unspliced and spliced dynamics, OPCs may have a faster RNA splicing process than MOLs to meet their immune functional demands at early and peak stages of the disease.

### Potential enhancers and DORCs

The intermodality investigation helped us associate detected peaks with the expression of the most probable nearby genes using the LinkPeaks function from Signac, inspired by the method described in the SHARE-seq paper^12^. Each peak-to-gene connection, occurring in at least ten cells and within a 50,000-bp distance from the gene TSS, with a positive Pearson correlation coefficient and a *P* value lower than 0.05 was considered a meaningful interaction. For each cell, the number of fragments falling into the 47,610 peaks involved in the same number of meaningful interactions was divided by the total number of fragments falling in peaks for the same cell. From the normalized matrix of meaningful peaks per cell, the normalized numbers of fragments in peaks were aggregated by gene using the peak-to-gene connections, for a total of 14,665 genes joined to at least one peak. The regulatory chromatin score for each cell of the generated genes was then multiplied by a scaling factor of 10,000. Notwithstanding the value of such enhancer scores, we were interested in deciphering more consequent DORCs. Therefore, we selected a pool of genes connected to at least five peaks to redo the peaks-to-genes connectivity calculation on a broader distance of 500,000 bp from the gene TSS. Keeping a positive Pearson correlation coefficient and a *P* value lower than 0.05, a total of 66,105 peaks were connected to a total of 3,369 genes defined as DORCs. Similar to the regulatory chromatin of genes, the DORCs were normalized, aggregated per gene according to the new connection material and scaled to generate a DORC score.

As calculating the sum of the peak counts conserves the negative binomial distribution of the epigenetic modality, both regulatory chromatin and DORC-regulated genes were reversed into unnormalized matrices (scaling factor and number of fragments in peaks for a given cell) to be loaded into a SingleCellExperiment object to ascertain, with confidence, gene variations between time points in a pseudobulk manner as described above, with the same parameters and thresholds.

### GRNs and predicted transcription factors

GRNs were inferred with Pando 1.0.0 (ref. ^31^). Pando models the relationship between transcription factors and their binding sites in selected regulatory regions with the expression of target genes combining multiome RNA and ATAC information.

The whole dataset was subset in MOL2 and MOL5/MOL6. For each MOL, we randomly subset a maximum of 2,000 cells per time point. We selected peak regions specific to each time point in MOL2 and MOL5/MOL6 (‘Multimodality features selection’) as candidate regulatory regions to scan for transcription factor binding motifs with JASPAR (2022 release).

The GRNs were inferred by fitting generalized linear models (GLMs) implemented in Pando for the expression of each gene. The regression model with peak–transcription factor pairs as independent variables and target genes as response variables was built for MOL2 and MOL5/MOL6 populations independently. Peaks were assigned to nearby genes with the peak_to_gene_method = ‘Signac’, which considers the closest distance, upstream or downstream, to the gene. The model estimates of each predicted transcription factor with a target gene can be interpreted as a measure of interaction between both. We calculated transcription factor activity by multiplying the mean estimates (coefficient) in the model by the average transcription factor expression at each time point. Transcription factor activities were ranked per the highest positive activity in each time point. Gene modules were selected with an *R*^2^ threshold of an adjusted *P* value lower than 0.05 and default parameters with the Pando find_modules function.

### IFNγ treatment, bulk RNA-seq and bulk ATAC-seq processing

A total of two replicates per condition were sequenced for both bulk RNA-seq and bulk ATAC-seq. Raw FastQ files were aligned and processed using the NextFlow core ATAC and RNA pipelines^90^, nextflow v24.04.2, to the genome assembly GRCm38.

For bulk RNA-seq, gene-level integer raw counts tables from nf-core Salmon output were loaded into R and used as input for edgeR v4.2.0 (refs. ^91,92^) together with their associated metadata. The combined counts table was used to estimate the library size, perform CalcNormFactors with the trimmed mean of M values (TMM) method and perform the fit of genewise common dispersion (glmFit), followed by a likelihood ratio test (glmLRT). Benjamini and Hochberg’s algorithm test was used to control the FDR.

First, differential gene expression was tested for changes in the first dose of IFNγ treatment versus control. Second, the resulting genes with a *P* value of <0.05 and FDR of <0.05 were tested for differential gene expression changes after the second IFNγ treatment dose. The experiment design included batch (replicates) and treatment as covariates. Visualization of the expression levels of differentially expressed genes was performed after running VST normalization from DESeq2 v1.44.0 in the whole dataset, including all replicates from the five time points. The final visualization shows the scaled *z* scores through the whole table, (scale function center = TRUE) for visualization purposes.

Aligned reads for treatment and replicates were extracted from processed BAM files from the nf-core ATAC-seq pipeline. Using BedTools v2.25.0 (ref. ^93^) coverage on the defined TSS windows and enhancer regions as a reference, an integer count table was built for enhancer regions and TSS windows, respectively, with defined regions as rows and samples as columns. Raw count tables on defined regions were loaded into R and used as input for edgeR v4.2.0, together with their associated metadata. Count tables were used to estimate the library size, perform CalcNormFactors with the TMM method and perform the fit of regionwise common dispersion (glmFit), followed by a likelihood ratio test (glmLRT). Differential accessibility testing was performed with the design (~0 + treatment, replicate) with makecontrast IFNγ 24 h (+0 h) – IFNγ 24 h (+96 h). Benjamini and Hochberg’s algorithm test was used to control the FDR. TSS windows and enhancer regions were selected based on a ⎸ log (FC) ⎸ of <0.5 and FDR of >0.05.

To define the TSS windows, also referred to as promoter regions, all annotated TSSs from EnsEmbl Mouse genes version 102 were extracted. Each TSS was extended 25 bp upstream and 25 bp downstream. The resulting resized TSS regions were merged whenever there were overlaps between them using BedTools 2.17.0 mergeBed –s –nms. The resulting merged TSS regions were resized, adding upstream 500 bp and downstream 500 bp from the center. To avoid repeated regions, each window was annotated with the closest gene as a consensus. Windows including more than one TSS usually referred to alternative TSSs of the same gene. One gene can have several TSS windows assigned. The final annotations consisted of 113,018 nonoverlapping TSS windows of 1 kb in length.

To define enhancer regions, the regions defined as proximal and distal enhancers from ENCODE candidate *cis*-regulatory element (cCRE; GRCm38/mm10) assemblies and last updated 26 May 2021 were retrieved. Enhancer cCREs overlapping the defined TSS windows (‘IFNγ treatment, bulk RNA-seq and bulk ATAC-seq processing’) were filtered out using BedTools v2.25.0 intersect –v. Resulting enhancer cCREs were merged in nonoverlapping regions with bedtools 2.17.0 mergeBed –s –nms and extended 500 bp upstream and downstream from the center. To avoid regions without evidence from our primary OPC bulk ATAC-seq analysis, any defined region not overlapping the consensus peaks from all conditions called by nf-ATACseq were discarded. To annotate enhancers to potentially regulated genes, each enhancer region was assigned to a candidate gene based on the previously calculated peaktogene interactions, CICERO, in our single-cell multiome data from OLG. The final annotations consisted of 59,857 nonoverlapping enhancer regions of 1 kb in length. One enhancer region can have several annotated cCREs and be assigned to different genes.

Visualization of ATAC signal on the TSS windows and the enhancer regions of the assigned genes was performed after running VST normalization from DESeq2 1.44.0 in the whole dataset, including all replicates from the five time points. Normalized counts were scaled from 0 to 1 by row (gene) for visualization purposes^94–96^

### Heat maps

Samples with less than 5 cells and time points with less than 30 cells were removed before heat map generation. For gene expression and gene/promoter accessibility, normalized log transform aggregated gene expression and gene/promoter accessibility at each time point and cell type were calculated to generate heat maps scaled across time points from 0 (low) to 1 (high). For gene regulatory chromatin, the average value of the gene regulatory chromatin score at each time point and cell type was scaled across time points from 0 (low) to 1 (high). Missing values in any tested modalities were set to 0. The black column on the right side of each heat map represents the gene average raw count. For each modality, the average of the previously calculated scaled values per gene category and time point was used to produce the summarized dynamic line plots and their associated standard deviation of the distribution shown as a band of the same color.

### GO

Highlights of biological pathways involving the most dynamic features along the disease time course were assessed using the biomaRt v2.58.2 R package in addition to the Ensembl v79_2.99.0 database. For each cell type, the top 50 features with the highest amplitude across time points were selected. Genes possessing an Entrez Gene ID underwent pathway enrichment via the ‘enrichPathway’ function from the ReactomePA v1.46.0 R package, with a pvalueCutoff set at 0.05, a qvalueCutoff set to 0.05 and FDR (‘fdr’) as a method of adjustment. A maximum of ten pathways (adjusted *P* value of <0.05) ranked by adjusted *P* value were displayed on dot plots. Network graphs were built on significant pathways (adjusted *P* value of <0.05) from the ‘emapplot’ function, taking as input the results of the pairwise_termsim function, with the Jaccard similarity coefficient as the calculation method for a maximum of 50 pathways ranked by adjusted *P* value.

### Genome tracks

Coverage of the DNA fragments within a given genomic region was determined using CoveragePlot from Signac. Tracks were normalized by group using a scaling factor as the number of cells within the group multiplied by the sequencing depth average of the group.

### Single-cell genomic heat maps

All fragments from 50 randomly selected cells per cluster and falling into a specific genomic region were carried into a 250-bp windowed matrix that was further down-binarized and plotted.

### Percentage of cells on stacked bar plots

The number of cells per cell subtype in each condition was aggregated, normalized across conditions to get a proportion of each cell subtype per condition and divided by the number of conditions to keep the sum of the proportion equal to 100%.

### Percentage of cells on side-by-side bar plots

The number of predicted male and female cells in each sex-specific sample was aggregated and normalized across samples to get a proportion of each sex per sample.

### Circos plots

Distribution of cell numbers in the top levels of the circos plots, matched to each corresponding bottom level. For an unbiased visualization of proportion, some circos plots were randomly downsampled by top levels, bottom levels or both (details are mentioned in the figure legends).

### Bigwig files

The mouse genome was segmented into 100-bp windows, and fragments were assigned to their corresponding window, generating a binarized genome per cell matrix. For each group of cells, the fragment counts matrix was aggregated, multiplied by a scaling factor of 10,000 and divided by the number of fragments in the group.

### Statistics and reproducibility

Data were processed inside notebooks within a singularity environment (‘Code availability’) on a high-performance computer cluster running under Ubuntu 20.04 LTS. One EAE early time point sample with a score of 0 was removed from the analysis due to no EAE symptoms and similar gene expression as CFA-Ctrl, as well as a MOL cluster specific at 97.86% for a unique sample at the early time point. Differential gene expression was determined using the DESeq2 package using negative binomial GLMs, which is the expected distribution from single-cell experiments^97,98^. Animals of both sexes were randomly assigned to experimental groups using the GraphPad randomization tool. The number of cells in each sample was selected to fit the specifications outlined by 10x Genomics. The investigators were not blinded to allocation during experiments and outcome assessment.

### Reporting summary

Further information on research design is available in the Nature Portfolio Reporting Summary linked to this article.

## Online content

Any methods, additional references, Nature Portfolio reporting summaries, source data, extended data, supplementary information, acknowledgements, peer review information; details of author contributions and competing interests; and statements of data and code availability are available at 10.1038/s41593-025-02100-3.

## Supplementary information

Supplementary Information Supplementary Methods.

Reporting Summary

Supplementary Table 1 Differential gene expression and chromatin accessibility, IFNγ doses, bulk RNA-seq and bulk ATAC-seq.

Supplementary Table 2 Marker features for sub-OLG populations.

Supplementary Table 3 Differential modality features between time points.

Supplementary Table 4 GO of differential modalities between time points.

Supplementary Table 5 Differential modality features between MOL5/MOL6 and MOL2.

Supplementary Table 6 GO of differential modalities between MOL5/MOL6 and MOL2.

Supplementary Table 7 Differentially expressed genes and accessibility in OLG populations.

Supplementary Table 8 GO of differentially expressed genes and accessibility in OLG populations.

Supplementary Table 9 Damage- and IFN-associated genes.

Supplementary Table 10 qPCR primer sequences.

## Source data

Source Data Figs. 2 and 8 and Extended Data Fig. 8 Statistical source data.

## Data Availability

Raw fastq files, counts matrices and genomic tracks are available on Gene Expression Omnibus (GSE250589, GSE283085 and GSE283086). Data are also available for browsing at the University of California, Santa Cruz, Cell Browser and Genome Browser (https://olg-dyn-eae-multiome.cells.ucsc.edu) and at https://ki.se/en/mbb/research/research-division-of-molecular-neurobiology/goncalo-castelo-branco-group/oligointernode. Source data are provided with this paper.

## Code availability

Jupyter notebooks to process the raw datasets and reproduce the figures are accessible at https://github.com/Castelo-Branco-lab/EAE_multiomics_2025.
