# Supplementary material for: Distinct transcriptomic and epigenomic responses of mature oligodendrocytes during disease progression in a mouse model of multiple sclerosis
Source: Nat Neurosci. 2025 Nov 17;28(12):2612–27. doi: 10.1038/s41593-025-02100-3 (PMC12672374; doi:10.1038/s41593-025-02100-3)
Supplement: Supplementary file 1 — Supplementary Methods. [file 41593_2025_2100_MOESM1_ESM.pdf]

nature neuroscience

Supplementary information

https://doi.org/10.1038/s41593-025-02100-3

Distinct transcriptomic and epigenomic responses of mature oligodendrocytes during disease progression in a mouse model of multiple sclerosis

In the format provided by the authors and unedited

Filter out low QC cells.

Depending on the quality of each sample, for each cell, a maximum of 30,000/150,000 and a minimum of 1,000 ATAC counts, a maximum of 10,000/50,000 and a minimum of 600 RNA counts, a minimum of 250 detected genes, a maximum of 0.8/1.5 nucleosomal signal, a TSS minimum enrichment of 2 and a maximum percentage of mitochondrial information of 15/50 were prerequisites to consider a given cell for the downstream analysis. These thresholds led to a reduction by around 12.6% the overall number of cells, from 179,078 down to 156,205 (Naïve-Ctrl 19,134 cells, CFA-Ctrl 49,274 cells, EAE early stage 37,576 cells, EAE peak stage 18,000 cells, EAE late stage 32,221 cells). While these thresholds are on purpose not too strained, they did not lead to any clustering perturbation downstream.

Peak connection. To consider a peak associated to a given gene, the function LinkPeaks from Signac was used, with pearson correlation method, a distance from the gene of 500,000bp maximum, a minimum of 10 cells possessing the peak and the expression of the given gene, a n_sample at 200 and a p-value cutoff at 0.05. The p-values were corrected by Benjamini-Hochberg method and only the association with a p-value adjusted lower than 0.01 were selected for downstream analysis. After rescaling the score to a maximum of 1, a total of 74,685 peak to gene connections were saved. To consider a peak associated to another peak, we downsampled the dataset to 1000 cells in each time point and we binarized the peak count matrix. Cicero v1.3.9 was applied to this matrix by the two functions make_cicero_cds and run_cicero, with the UMAP coordinated inputted to the first function and the genomic coordinates of our mouse genome to the second function. Cicero generated 17M of putative connections between peaks. A p-value was calculated for each of them based on the zscore of the coaccessibility score, for both tails of the distribution. These values were adjusted by Benjamini-Hochberg method and connections with an adjusted p-value lower than 0.01 were selected. After rescaling the score to a maximum of 1, a total of 285,034 peak to peak connections were saved. The aggregation of peak to gene and peak to peak connections were recorded in a bedpe file, similar to the cellranger-arc output.

Doublet detection. The detection of putative events where more than one cell in the same droplet occurs was done using DoubletFinder v2.0.4[79]. The gene counts matrix for individual sample was normalized by library size, multiplied by 10,000 and natural-log transformed. The expression of the top 1,000 most variable genes was centered by subtracting their average expression, scaled by dividing their standard deviation, and used for a Principal Component Analysis (PCA). The Euclidean distances between each cell were calculated using the first 15 Principal Components (PCs) to build a Shared k-Nearest Neighbors (SNN) graph. A Louvain algorithm was used to optimize the modularity of the SNN graph to determine clusters at

a resolution of 0.5. Then a Uniform Manifold Approximation and Projection (UMAP) dimensional reduction was applied to the first 15 PCs to project the cells on a 2D space.

The pN-pK parameter sweep was performed with the paramSweep_v3 function on the first 15 PCs to a maximum of 10,000 cells and then pass on the summarize Sweep function to summarize and compute the bimodality coefficient across pN and pK parameter space. The mean-variance normalized of the bimodality coefficient was computed using the find.pK function to highlight the optimum pK value of each sample. The best pK value of each sample was fluctuating between 0.005 and 0.25. While the number of generated artificial doublets (pN) was set to 0.25 as a default parameter for all samples, the prediction of the proportion of the total number of doublets (nExp) per sample was estimated by creating a model of the proportion of homotypic doublets via the annotated clusters mentioned above. The homotypic proportion complement was then multiplied by the number of cells in the sample times the best pK of the same sample. The first 15 PCs, the pN, best pK, and nExp were set as parameters in the doubletFinder_v3 function to calculate the proportion of Artificial Nearest Neighbors (pANN) for each real cell. This pANN score is then compared to the number of expected doublets to generate the final doublet predictions.

Most of the samples drawn less than 1% of doublets but 8 samples drawn 1.49%, 1.59%, 1.88%, 2.35%, 3.33%, 5.08%, 7.85% and 20.86% of doublets. Using the multiplet rate provided by 10X genomics (https://kb.10xgenomics.com/hc/en-us/articles/360001378811-What-is-the-maximum-number-of-cells-that-can-be-profiled-) we were able to measure a 0.8%/8% theoretical number of doublets. Predicted doublets were removed from the downstream analysis.

Sex classification model. A total of 69,152 cells were sequenced with a priori sex annotation (37,980 cells were female and 31,172 cells were male) from EAE time points (16,699 cell from control, 28,974 cells from early, 8368 cells from peak and 15,111 cells from late stage). After doublets removal, only 67,732 remain. This knowledge was used to create a machine-learning sex classifier to investigate sex transcriptomic or epigenetic differences. An equal number of cells for each gender (30,940 cells) were extracted from the main object to a sex object to build the model. Mouse genes and positions from chrX and chrY were extracted from Ensembl v79_2.99.0[76]. For each chromosome and each cell, the sum of the gene count expression of those existing in the sex object was divided by the total number of counts by cell, generating a percentage of reads falling into transcribable regions on either chrX or chrY. In addition, a gene score was calculated from *Xist* using the AddModuleScore from Seurat, with 200 control features from the same bin per analyzed feature. The sex object was then again subdivided into a training dataset and a testing dataset, representing 80% and 20% of the dataset, respectively. In association with the sex

annotation, these three scores were sufficient to train a random forest classifier from the Caret v6.0 package in R, able to differentiate male and female cells with high accuracy.

The random forest function was run using the train function in Caret. The trControl parameter, for controlling the computational nuances of the main function, was calculated using the trainControl function with "cv" method, 5 number of folds, derivating from the createFolds function on the sex annotation to 5 folds, asking for a "grid" search and to class the probabilities turning on the "classProbs" parameter, saving the final predictions in two classes. The tuneGrib parameter was set to consider the number of variables to randomly sample (mtry) as 1 and 2. The model was trained sequentially on different node sizes (20, 30 and 50) and number of trees (5,000, 10,000 and 20,000). The remaining parameters were set as default and the ROC value calculation was turned on to assess the best node size, number of trees and mtry combo. Not by far, the association of 5,000 trees and 50 nodes and 1 mtry outperformed the other combinations.

The validation on the testing dataset yielded an accuracy of 95.4%, a sensitivity of 91.0% and a specificity of 99.7%. An insignificant number of male cells were annotated as female cells, while a few female cells were assigned to male cells. We then used the newly created model to process each cell of the main object. Correction of miss assigned cells (8.32%) from a priori sex annotated samples was performed.

Some studies have reported that immune responses are different in many aspects between males and females in both MS and its animal models[80, 81]. Since most of our samples were mixed with one male mouse and one female mouse, we created a sex prediction model based on the expression of sex-related genes (see methods). This sex prediction model was validated with samples containing cells from only male or female EAE mice, with an accuracy of 95.3% on the validation dataset. There was better prediction accuracy towards the male sample (99.48%) than in the female sample (86.13%) (**Extended Data Fig. 9b,c**). We applied this sex prediction model to the entire dataset, annotated the sex of the cells (**Extended Data Fig. 9d,e**), and compared the differences between males and females OLGs based on the sex prediction results. For OLG sub-populations, apart from sex related genes, such as X inactive specific transcript (*Xist* and *Tsix),* ubiquitously transcribed tetratricopeptide repeat containing Y-linked (*Uty*)*,* eukaryotic translation initiation factor 2 subunit 3 structural gene Y-linked *(Eif2s3y*)*,* dead-box helicase 3 Y-linked (*Ddx3y*)*,* and lysine demethylase 5D (*Kdm5d*), no other genes were found differentially expressed between male and female (**Extended Data Fig. 9f**). Accordingly, we found no major differences between the OLGs of males and females at the gene expression level in our data. We also did not observe a significant difference in the percentages of imOLGs between male and female mice (**Extended Data Fig. 9f-h**). Thus, our data indicates

that the OLG response to the neuroinflammatory environment in EAE is not characterized by sexual dimorphism.

OL lineage investigation. All cells belonging to the OL lineage were subsetted for fine-tuning annotation. One EAE early time point sample collected on day 8 post-immunization with a score of 0 (without any symptom but with weight loss during the disease course) was removed from the analysis due to no EAE symptom and similar gene expression as CFA-Ctrl. As no major gene expression differences were found between Naïve-Ctrl and CFA-Ctrl in OL lineage population, Naïve-Ctrl samples were removed from the downstream analysis. The processing of this OL lineage subset of 120,183 cells, is similar to the one with all cell types. Nevertheless, few differences must be mentioned, the 20 first PCs and the first 9 LSIs were selected for graphs construction. Last, cluster resolutions 2.2, 0.8 and 3.6 were selected for gene expression, peak accessibility and joined modality respectively. An in-depth label transfer was performed on the subset with the same methods as previously described, using only the OL lineage cells from the literature dataset[4] **(Extended Data Fig. 3c,d)**. From the 47 potential OL lineage cells clusters on joined graph clustering resolution, we aggregated them using different hierarchical clustering methods (single, complete, average, mcquitty, centroid, median, ward.D and ward.D2) based on the scaled aggregated expression of the top 20 highly variable genes in each cluster selected by Wilcoxon Rank Sum test using FindAllMarkers function from Seurat (p-value adjusted lower than 0.05, average log2 Fold Change higher than 0.5 and percentage of cells expressing a gene superior than 50%). For each method, the arbitrary scission of COPs and COPs into two individual cluster was set as a height threshold for cluster aggregation. Each method yielded different number of aggregated clusters (single: 5, complete: 20, average: 18, mcquitty: 18, centroid: 15, median: 14, ward.D: 20 and ward.D2: 19). The selection of the most suitable hierarchical clustering method for this dataset was done by testing the aggregated clusters technical robustness across the different methods by calculating a Jaccard similarity of cell names to all cluster's combination. The goal of this comparison was to highlight clusters specific to a sole method and those consistent across methods. The biological robustness was also assessed by comparing the list of differential gene expression between all original clusters inside each aggregated clusters by Wilcoxon Rank Sum test using FindAllMarkers function from Seurat (p-value adjusted lower than 0.05, average log2 Fold Change superior than 0.5 and percentage of cells expressing a gene superior than 50%). The goal of this comparison was to highlight gene expression variation within each aggregated cluster, not to under cluster some clusters. With these two metrics, the average method of hierarchical clustering was selected to aggregate the clusters . The OPC cluster was subdivided into three sub clusters as we witnessed two time point specific groups of OPCs and a third one expressing cycling genes. Each aggregated cluster was manually assigned to the main OL lineage cell types

(OPC, COP, MOL1, MOL2, MOL5/6) using specific gene markers and label transfers. OL lineage cell type sub clustering (α, β, γ, δ, ε, ζ, η, θ, ι, κ) was assessed and ordered along their average immune score for each main OL lineage cell type **(Extended Data Fig. 4a)**. For instance, when compared to other MOL2 populations, MOL2-ε, predominantly derived from the peak stage of EAE (Fig. 3c), exhibited an enrichment of immune-related genes, including Interferon Induced Protein With Tetratricopeptide Repeats 3 (Ifit3), Nlrc5, and Interferon Gamma Induced GTPase (Igtp), among others (Supplementary Table - Tab2). MOL2-α, which mainly came from CFA-Ctrl and EAE at the early stage, expressed higher lipid metabolic process genes, such as 3-Hydroxy-3-Methylglutaryl-CoA Synthase 1 (Hmgcs1), Cytochrome P450 Family 27 Subfamily A Member 1 (Cyp27a1), and Squalene Epoxidase (Sqle) (Supplementary Table - Tab2). Oligodendrocytic Myelin Paranodal And Inner Loop Protein (Opalin), which is associated with oligodendrocyte differentiation, was enriched in MOL5/6-β. A significant increase in the expression of actin cytoskeleton organization related genes, such as Actin Binding LIM Protein Family Member 2 (Ablim2), NCK Associated Protein 5 (Nckap5), and Prickle Planar Cell Polarity Protein 1 (Prickle1), were observed in MOL5/6-ζ compared to other MOL5/6 populations (Supplementary Table - Tab2). The majority of MOL2-β and γ were derived from EAE late stage (Fig. 3b,c), with gene markers associated with metabolism and differentiation like Ectonucleotide Pyrophosphatase/Phosphodiesterase 6 (Enpp6) and S100 Calcium Binding Protein B (S100b) (Supplementary Table - Tab2). In addition, the majority of the cells MOL5/6-α, β, and γ were mainly comprised of cells from CFA-Ctrl and EAE at the early stage, MOL5/6-θ were mainly comprised of cells from EAE at the peak stage and cells from MOL5/6-ζ and ι were drawn by EAE at the late stage (Fig. 3b,c).
