# Supplementary material for: Distinct transcriptomic and epigenomic responses of mature oligodendrocytes during disease progression in a mouse model of multiple sclerosis
Source: Nat Neurosci. 2025 Nov 17;28(12):2612–27. doi: 10.1038/s41593-025-02100-3 (PMC12672374; doi:10.1038/s41593-025-02100-3)
Supplement: Supplementary file 2 — Reporting Summary [file 41593_2025_2100_MOESM2_ESM.pdf]

nature portfolio

Corresponding author(s): Gonçalo Castelo-Branco

Last updated by author(s): Aug 6, 2025

Reporting Summary

Nature Portfolio wishes to improve the reproducibility of the work that we publish. This form provides structure for consistency and transparency in reporting. For further information on Nature Portfolio policies, see our Editorial Policies and the Editorial Policy Checklist.

Statistics

For all statistical analyses, confirm that the following items are present in the figure legend, table legend, main text, or Methods section.

n/a	Confirmed	
☐	☒	The exact sample size (*n*) for each experimental group/condition, given as a discrete number and unit of measurement
☐	☒	A statement on whether measurements were taken from distinct samples or whether the same sample was measured repeatedly
☐	☒	The statistical test(s) used AND whether they are one- or two-sided<br>*Only common tests should be described solely by name; describe more complex techniques in the Methods section.*
☐	☒	A description of all covariates tested
☐	☒	A description of any assumptions or corrections, such as tests of normality and adjustment for multiple comparisons
☐	☒	A full description of the statistical parameters including central tendency (e.g. means) or other basic estimates (e.g. regression coefficient) AND variation (e.g. standard deviation) or associated estimates of uncertainty (e.g. confidence intervals)
☐	☒	For null hypothesis testing, the test statistic (e.g. *F*, *t*, *r*) with confidence intervals, effect sizes, degrees of freedom and *P* value noted<br>*Give P values as exact values whenever suitable.*
☒	☐	For Bayesian analysis, information on the choice of priors and Markov chain Monte Carlo settings
☒	☐	For hierarchical and complex designs, identification of the appropriate level for tests and full reporting of outcomes
☐	☒	Estimates of effect sizes (e.g. Cohen's *d*, Pearson's *r*), indicating how they were calculated

Our web collection on statistics for biologists contains articles on many of the points above.

Software and code

Policy information about availability of computer code

Data collection	Single cell multiome data was collected using Illumina NovaSeq 6000 (NovaSeq Control Software 1.7.5/RTA v3.4.4). Raw fastq files were aligned for both ATAC and RNA to mm10 genome associated with Cellranger ARC2 v2.0.2 (GENCODE vM23/Ensembl98), with default settings and aggregated using aggr function from the same software without normalization. Bulk-RNA-seq and bulk ATAC-seq data were collected using Illumina NovaSeq X with standard software. Raw fastq files were aligned and processed using NextFlow v24.04.2 core ATAC and RNA pipelines to the genome assembly GRCm38. Images acquired using a Zeiss LSM800 Confocal (RNAscope) and Zeiss LSM980 (immunohistochemistry). OPC cellular fluorescence was measured with CantoII (BD Biosciences).
Data analysis	Single cell multiomics data was analyzed using mainly R v.4.3.2, Seurat v5.0.3 and Signac v1.13.0 packages. Ambiant RNA was removed using Cellbender v0.3.0 and potential doublets were called using DoubletFinder v2.0.4.Peaks were called using MACS2 v3.0.0 and linked to each others genes using Cicero v1.3.9. The sex determination was created using a random forest model from Caret v6.0. The Local Inverse Simpson's Index (LISI) was performed using Harmony v1.2.0. Differential features expression or accessibility were computed using SingleCellExperiment v1.24.0 and DESeq2 v1.42.1. Gene Ontology (GO) was assessed using ReactomePA v1.46.0. Genes velocity were processed from velocyto v.0.17.17 and MultiVelo v.0.1.3. Gene Regulatory Network (GRN) was generated using Pando v1.0.0.<br>Bulk RNA-seq and ATAC-seq were analysed with the NextFlow v24.04.2 nf-core RNA-seq and ATAC-seq pipelines. Differential features expression or accessibility were processed using edgeR v4.2.0 and DESeq2 v1.44.0. Count tables were built for enhancer regions and TSS windows using BedTools v2.25.0. All code to reproduce the analysis step by step is published in notebooks available at : https://github.com/Castelo-Branco-lab/EAE_multiomics_2025. RNAscope and immunohistochemistry images were processed in Fiji/ImageJ (1.54f). Scores for EAE and CFA-Ctrl were plotted using GraphPad Prism version 9.0.0. Flow cytometry data were analyzed with FlowJo software 10.8.1 (TreeStar).

For manuscripts utilizing custom algorithms or software that are central to the research but not yet described in published literature, software must be made available to editors and reviewers. We strongly encourage code deposition in a community repository (e.g. GitHub). See the Nature Portfolio guidelines for submitting code & software for further information.

Data

Policy information about availability of data

All manuscripts must include a data availability statement. This statement should provide the following information, where applicable:

- Accession codes, unique identifiers, or web links for publicly available datasets
- A description of any restrictions on data availability
- For clinical datasets or third party data, please ensure that the statement adheres to our policy

Mouse reference genome associated with Cellranger ARC2 v2020-A-2.0.0 (GENCODE vM23/Ensembl98) was used for the single cell multiomics. For the bulk RNA-seq and ATAC-seq, the mouse reference genome GRCm38 was used.
From the single cell dataset, Ensembl base annotation v79_2.99.0 was used to annotated peaks. Gene Ontology database was provided by biomaRt v2.58.2.
Cells association with immune response were characterize using GO:0002250, with immune system process using GO:0002376 and the damage-associated from Kaya, T., et al., Nat Neurosci, 2022 Supplementary Table3. Finally RepeatMasker database whas queried from https://genome.ucsc.edu with the following options : Mouse genome, GRCm38/mm10 Dec2011, Group by All Track, RepeatMasker Track for the whole genome output as GTF file, in order to consider repeat elements and low complexity sequences.
For the bulk dataset, JASPAR 2022 database, was used to find Transcription Factors (TFs) binding motifs and ENCODE Candidate Cis-Regulatory Elements (cCREs) (GRCm38/mm10) assembly (last update on 2021-05-26) was used to define enhancers.
Raw and processed data are available under GSE250589. GSE283085 (ATAC-seq) and GSE283086 (RNA-seq).
The following publicly available dataset was used in this study for label transfer GSE113973 (scRNA-seq of oligodendrocytes in EAE).
Statistical source data for image quantification, qPCR, and flow cytometry analyses are provided in the accompanying Source Data file.

Research involving human participants, their data, or biological material

Policy information about studies with human participants or human data. See also policy information about sex, gender (identity/presentation), and sexual orientation and race, ethnicity and racism.

Reporting on sex and gender	n/a. No human data is included in this study.
Reporting on race, ethnicity, or other socially relevant groupings	n/a. No human data is included in this study.
Population characteristics	n/a. No human data is included in this study.
Recruitment	n/a. No human data is included in this study.
Ethics oversight	n/a. No human data is included in this study.

Note that full information on the approval of the study protocol must also be provided in the manuscript.

Field-specific reporting

Please select the one below that is the best fit for your research. If you are not sure, read the appropriate sections before making your selection.

☒ Life sciences ☐ Behavioural & social sciences ☐ Ecological, evolutionary & environmental sciences

For a reference copy of the document with all sections, see nature.com/documents/nr-reporting-summary-flat.pdf

Life sciences study design

All studies must disclose on these points even when the disclosure is negative.

Sample size

No sample size calculation was performed to pre-determine sample sizes. For all experiments, a minimum of three biological replicates per condition was used. Although no a priori statistical power calculation was performed, sample sizes were selected based on precedent in the literature and our prior experience with similar experimental designs, where n ≥ 3 has consistently yielded reproducible and statistically discernible effects.

EAE samples:
- 7 multiome experiments for the early stage (7 females plus 7 male mice, total 14 mice)
- 5 multiome experiments for the peak stage (5females plus 5 male mice, total 10 mice)
- 6 multiome experiments for the late stage (7 females plus 7 male mice, total 14 mice)

CFA-CTRL samples:

- 5 multiome experiments for the early stage (5 females plus 5 male mice, total 10 mice)
- 2 multiome experiments for the peak stage (2 females plus 2 male mice, total 4 mice)
- 2 multiome experiments for the late stage (2 females plus 2 male mice, total 4 mice)

3 multiome experiments were performed for Naïve-Ctrl (3 females plus 3 male mice, total 6 mice).

For RNAscope ISH and immunohistochemistry, the sample size was n=3 per condition. For RNAscope ISH, six 20X randomly selected fields per mouse (three from lesion and three from non-lesion) were chosen for quantification.

Data exclusions

For single-cell multiome data, we excluded data points through our quality control pipeline, as indicated in the methods section in the paper. In short, depending on the quality of each sample, for each cell, a maximum of 30,000/150,000 and a minimum of 1,000 ATAC counts, a maximum of 10,000/50,000 and a minimum of 600 RNA counts, a minimum o 250 detected genes, a maximum of 0.8/1.5 nucleosomal signal, a TSS minimum enrichment of 2 and a maximum percentage of mitochondrial information of 15/50 were prerequisites to consider a given cell for the downstream analysis. One EAE early time point sample collected on day 8 post-immunization with a score of 0 was removed from the analysis due to no EAE symptom and similar gene expression as CFA-Ctrl. A cluster of 1973 cells specific at 97.86% to one Early sample, was removed from the analysis.

For the bulk RNA-Seq and ATAC-Seq experiments, for each treatment and control data 3 replicates were collected and sequenced. After preprocessing and QC with RNA-seq nf-core pipeline QC statistics, one of the replicates from the second dose of IFN-g treatment of one of the replicates showed significant differences compared to the other replicates, based on PCA inspection of similarity between replicates and Euclidean distance between replicates between others. For consistency reasons, this replicate from all the treatments and control was discarded. All downstream analyses were performed with 2 replicates for both RNA-seq and ATAC-seq in all data points.

Replication

RNAscope ISH and immunohistochemistry were performed with n = 3 independent biological replicates per condition. Multiome experiments included 7 (14 mice) early-stage, 5 (10 mice) peak-stage, and 6 (14 mice) late-stage EAE experiments; 5 (10 mice), 2 (4 mice), and 2 (4 mice) CFA-control experiments at early, peak, and late stages respectively; and 3 (6 mice) naïve controls, all independently replicated successfully. IFN-γ treatments were performed with n = 2 primary OPCs and n = 3 Oli-neu samples. Stat3 siRNA experiments included n = 5 for qPCR and n = 4 for flow cytometry, and Stat3 inhibitor treatments were done with n = 6, with all replication attempts successful.

Randomization

For single-cell RNA-seq, we distributed females and males with similar ages equally in controls and EAE, using the GraphPad randomization tool (GraphPad by Dotmatics). For cell experiments, cells used within each independent experiment were derived from the same passage or from the same animals, and therefore allocation to experimental groups was not randomized.

Blinding

The analysis involving RNAscope ISH were performed blindly. Blinding was not performed for EAE experiments, as disease monitoring and stage-specific tissue collection required knowledge of group allocation. For cell experiments, blinding was also not performed, as investigators were responsible for administering specific treatments to designated groups.

Behavioural & social sciences study design

All studies must disclose on these points even when the disclosure is negative.

Study description

Briefly describe the study type including whether data are quantitative, qualitative, or mixed-methods (e.g. qualitative cross-sectional, quantitative experimental, mixed-methods case study).

Research sample

State the research sample (e.g. Harvard university undergraduates, villagers in rural India) and provide relevant demographic information (e.g. age, sex) and indicate whether the sample is representative. Provide a rationale for the study sample chosen. For studies involving existing datasets, please describe the dataset and source.

Sampling strategy

Describe the sampling procedure (e.g. random, snowball, stratified, convenience). Describe the statistical methods that were used to predetermine sample size OR if no sample-size calculation was performed, describe how sample sizes were chosen and provide a rationale for why these sample sizes are sufficient. For qualitative data, please indicate whether data saturation was considered, and what criteria were used to decide that no further sampling was needed.

Data collection

Provide details about the data collection procedure, including the instruments or devices used to record the data (e.g. pen and paper, computer, eye tracker, video or audio equipment) whether anyone was present besides the participant(s) and the researcher, and whether the researcher was blind to experimental condition and/or the study hypothesis during data collection.

Timing

Indicate the start and stop dates of data collection. If there is a gap between collection periods, state the dates for each sample cohort.

Data exclusions

If no data were excluded from the analyses, state so OR if data were excluded, provide the exact number of exclusions and the rationale behind them, indicating whether exclusion criteria were pre-established.

Non-participation

State how many participants dropped out/declined participation and the reason(s) given OR provide response rate OR state that no participants dropped out/declined participation.

Randomization

If participants were not allocated into experimental groups, state so OR describe how participants were allocated to groups, and if allocation was not random, describe how covariates were controlled.

Ecological, evolutionary & environmental sciences study design

All studies must disclose on these points even when the disclosure is negative.

Study description	*Briefly describe the study. For quantitative data include treatment factors and interactions, design structure (e.g. factorial, nested, hierarchical), nature and number of experimental units and replicates.*
Research sample	*Describe the research sample (e.g. a group of tagged Passer domesticus, all Stenocereus thurberi within Organ Pipe Cactus National Monument), and provide a rationale for the sample choice. When relevant, describe the organism taxa, source, sex, age range and any manipulations. State what population the sample is meant to represent when applicable. For studies involving existing datasets, describe the data and its source.*
Sampling strategy	*Note the sampling procedure. Describe the statistical methods that were used to predetermine sample size OR if no sample-size calculation was performed, describe how sample sizes were chosen and provide a rationale for why these sample sizes are sufficient.*
Data collection	*Describe the data collection procedure, including who recorded the data and how.*
Timing and spatial scale	*Indicate the start and stop dates of data collection, noting the frequency and periodicity of sampling and providing a rationale for these choices. If there is a gap between collection periods, state the dates for each sample cohort. Specify the spatial scale from which the data are taken*
Data exclusions	*If no data were excluded from the analyses, state so OR if data were excluded, describe the exclusions and the rationale behind them, indicating whether exclusion criteria were pre-established.*
Reproducibility	*Describe the measures taken to verify the reproducibility of experimental findings. For each experiment, note whether any attempts to repeat the experiment failed OR state that all attempts to repeat the experiment were successful.*
Randomization	*Describe how samples/organisms/participants were allocated into groups. If allocation was not random, describe how covariates were controlled. If this is not relevant to your study, explain why.*
Blinding	*Describe the extent of blinding used during data acquisition and analysis. If blinding was not possible, describe why OR explain why blinding was not relevant to your study.*

Did the study involve field work? ☐ Yes ☐ No

Field work, collection and transport

Field conditions	*Describe the study conditions for field work, providing relevant parameters (e.g. temperature, rainfall).*
Location	*State the location of the sampling or experiment, providing relevant parameters (e.g. latitude and longitude, elevation, water depth).*
Access & import/export	*Describe the efforts you have made to access habitats and to collect and import/export your samples in a responsible manner and in compliance with local, national and international laws, noting any permits that were obtained (give the name of the issuing authority, the date of issue, and any identifying information).*
Disturbance	*Describe any disturbance caused by the study and how it was minimized.*

Reporting for specific materials, systems and methods

We require information from authors about some types of materials, experimental systems and methods used in many studies. Here, indicate whether each material, system or method listed is relevant to your study. If you are not sure if a list item applies to your research, read the appropriate section before selecting a response.

Materials & experimental systems

n/a	Involved in the study	
☐	☒	Antibodies
☐	☒	Eukaryotic cell lines
☒	☐	Palaeontology and archaeology
☐	☒	Animals and other organisms
☒	☐	Clinical data
☒	☐	Dual use research of concern
☒	☐	Plants

Methods

n/a	Involved in the study	
☒	☐	ChIP-seq
☐	☒	Flow cytometry
☒	☐	MRI-based neuroimaging

Antibodies

Antibodies used	MHCII (1:50, Invitrogen, 14-5321-85, Clone M5/114.15.2), GFP (1:200, Abcam, ab 13970), Goat anti-Rat Secondary Antibody(1:1000, Invitrogen, A21434), Goat anti-Chicken Secondary Antibody(1:1000, Abcam, ab150169), and PD-L1-APC-conjugated antibody (1:100, BioLegend, 124312, Clone 10F.9G2)
Validation	All antibodies used in this study are broadly used in the field and have been tested by the company.<br>-MHCII (Invitrogen, 14-5321-85, rat) : Host/Isotype Rat / IgG2b, kappa; applications: Western blot, immunohistochemistry , IHC on paraffin and frozen sections, immunocytochemistry/immunofluorescence, flow cytometry, ELISA, immunoprecipitation, neutralization, functional assays, inhibition assays, blocking assays, and in vitro assays. https://www.thermofisher.com/antibody/product/MHC-Class-II-I-A-I-E-Antibody-clone-M5-114-15-2-Monoclonal/14-5321-82<br>-GFP (Abcam, ab 13970, chicken): Host/Isotype: Chicken/IgY; applications: Western blot and immunocytochemistry/immunofluorescence. https://www.abcam.com/en-us/products/primary-antibodies/gfp-antibody-ab13970

Eukaryotic cell lines

Policy information about cell lines and Sex and Gender in Research

Cell line source(s)	Oli-neu Mouse Oligodendroglial Precursor Cell Line (RRID:CVCL_IZ82, obtained from Dr. Jacqueline Trotter, Johannes Gutenberg University, Germany).
Authentication	Cell line was not specifically authenticated but was used in genomic studies that were consistent with their identity.
Mycoplasma contamination	Cell line was regularly checked for mycoplasma contaminations and was found negative.
Commonly misidentified lines (See ICLAC register)	Oli�neu is not listed as a misidentified cell line.

Palaeontology and Archaeology

Specimen provenance	n/a
Specimen deposition	n/a
Dating methods	n/a

☐ Tick this box to confirm that the raw and calibrated dates are available in the paper or in Supplementary Information.

Ethics oversight	n/a

Note that full information on the approval of the study protocol must also be provided in the manuscript.

Animals and other research organisms

Policy information about studies involving animals; ARRIVE guidelines recommended for reporting animal research, and Sex and Gender in Research

Laboratory animals	Sox10:Cre-RCE:LoxP (EGFP) transgenic mice between 9-13 weeks were used in this study. Sox10:Cre-RCE:LoxP (EGFP) mice are a strain of mice obtained originally by crossing mice with Cre recombinase under the control of the Sox10 promoter (The Jackson Laboratories; with a C57BL/6 genetic background) with reporter mice RCE:loxP-EGFP (with CD1 background).
Wild animals	No wild animals were used in this study.
Reporting on sex	Both male and female mice were included in this study.
Field-collected samples	No field-collected samples were used in this study.
Ethics oversight	All experimental procedures on animals were performed following the European Directive 2010/63/EU, local Swedish directive L150/SJVFS/2019:9, Saknr L150, and Karolinska Institutet complementary guidelines for procurement and use of laboratory animals, Dnr. 1937/03-640 and Karolinska Institutet Comparative Medicine veterinary guidelines and plans (version 2020/12/18). The procedures described were approved by the local committee for ethical experiments on laboratory animals in Sweden (Stockholms Norra Djurförsöksetiska nämnd), license numbers: 1995-2019 and 7029-2020.

Note that full information on the approval of the study protocol must also be provided in the manuscript.

Clinical data

Policy information about clinical studies

All manuscripts should comply with the ICMJE guidelines for publication of clinical research and a completed CONSORT checklist must be included with all submissions.

Clinical trial registration	n/a
Study protocol	n/a
Data collection	n/a
Outcomes	n/a

Dual use research of concern

Policy information about dual use research of concern

Hazards

Could the accidental, deliberate or reckless misuse of agents or technologies generated in the work, or the application of information presented in the manuscript, pose a threat to:

No	Yes	
☒	☐	Public health
☒	☐	National security
☒	☐	Crops and/or livestock
☒	☐	Ecosystems
☒	☐	Any other significant area

Experiments of concern

Does the work involve any of these experiments of concern:

No	Yes	
☒	☐	Demonstrate how to render a vaccine ineffective
☒	☐	Confer resistance to therapeutically useful antibiotics or antiviral agents
☒	☐	Enhance the virulence of a pathogen or render a nonpathogen virulent
☒	☐	Increase transmissibility of a pathogen
☒	☐	Alter the host range of a pathogen
☒	☐	Enable evasion of diagnostic/detection modalities
☒	☐	Enable the weaponization of a biological agent or toxin
☒	☐	Any other potentially harmful combination of experiments and agents

Plants

Seed stocks	n/a
Novel plant genotypes	n/a
Authentication	n/a

ChIP-seq

Data deposition

☐ Confirm that both raw and final processed data have been deposited in a public database such as GEO.

☐ Confirm that you have deposited or provided access to graph files (e.g. BED files) for the called peaks.

Data access links<br>*May remain private before publication.*	n/a
Files in database submission	n/a
Genome browser session<br>(e.g. UCSC)	n/a

Methodology

Replicates	n/a
Sequencing depth	n/a
Antibodies	n/a
Peak calling parameters	n/a
Data quality	n/a
Software	n/a

Flow Cytometry

Plots

Confirm that:

☒ The axis labels state the marker and fluorochrome used (e.g. CD4-FITC).

☒ The axis scales are clearly visible. Include numbers along axes only for bottom left plot of group (a 'group' is an analysis of identical markers).

☒ All plots are contour plots with outliers or pseudocolor plots.

☒ A numerical value for number of cells or percentage (with statistics) is provided.

Methodology

Sample preparation	Mouse spinal cords were collected. Spinal cord tissues were then dissociated into a single cell suspension according to the manufacturer's protocol of Adult Brain Dissociation Kit, mouse and rat (Miltenyi Biotec, 130-107-677, we did not perform the red blood cells removal step since the majority of the red blood cells had been removed with PBS perfusion)
Instrument	BD FACS Aria III Cell Sorter was used for sorting. BD CantoII was used for analysis.
Software	FlowJo_v10.8.1 was used for analysis.
Cell population abundance	The percentages of GFP+ cells out of live cells differ across samples (1-15%), with EAE samples showed lower percentage compared to control. The percentages of PD-L1+ cells out of live cells differ across samples with different treatments (7.65-36.5%).
Gating strategy	Cells were identified first on FSC/SSC plots, dead cells were gated away using DAPI staining (sorting) or LIVE/DEAD Fixable Near-IR Dead Cell Stain Kit (analysis). For cell sorting, live cells were plotted on GFP (FITC channel was used) for collecting the GFP+ population. For PD-L1 expression analysis, live cells were plotted on APC channel for detecting PD-L1 expression.

☒ Tick this box to confirm that a figure exemplifying the gating strategy is provided in the Supplementary Information.

Magnetic resonance imaging

Experimental design

Design type	n/a

Design specifications | n/a

Behavioral performance measures | n/a

Acquisition

Imaging type(s) | n/a

Field strength | n/a

Sequence & imaging parameters | n/a

Area of acquisition | n/a

Diffusion MRI ☐ Used ☐ Not used

Preprocessing

Preprocessing software | n/a

Normalization | n/a

Normalization template | n/a

Noise and artifact removal | n/a

Volume censoring | n/a

Statistical modeling & inference

Model type and settings | n/a

Effect(s) tested | n/a

Specify type of analysis: ☐ Whole brain ☐ ROI-based ☐ Both

Statistic type for inference (See Eklund et al. 2016) | n/a

Correction | n/a

Models & analysis

n/a	Involved in the study
☐	☐ Functional and/or effective connectivity
☐	☐ Graph analysis
☐	☐ Multivariate modeling or predictive analysis

Functional and/or effective connectivity | n/a

Graph analysis | n/a

Multivariate modeling and predictive analysis | n/a
